# Supplementary material for: Visualizing the multi-level assembly structures of conjugated molecular systems with chain-length dependent behavior
Source: Nat Commun. 2023 Jun 7;14:3340. doi: 10.1038/s41467-023-39133-w (PMC10247739; doi:10.1038/s41467-023-39133-w)
Supplement: Supplementary file 1 — Supplementary Information [file 41467_2023_39133_MOESM1_ESM.pdf]

Supplementary Information

for

Visualizing the multi-level assembly structures of conjugated molecular systems with chain-length dependent behavior

Yang-Yang Zhou[1], Yu-Chun Xu[1], Ze-Fan Yao[1], Jia-Ye Li[1], Chen-Kai Pan[1], Yang Lu[1], Chi-Yuan Yang[1], Li Ding[1], Bu-Fan Xiao[1], Xin-Yi Wang[1], Yu Shao[1], Wen-Bin Zhang[1], Jie-Yu Wang[1], Huan Wang[1], and Jian Pei*[,1]

[1]Beijing National Laboratory for Molecular Sciences (BNLMS), Key Laboratory of Polymer Chemistry and Physics of Ministry of Education, Center or Soft Matter Science and Engineering, College of Chemistry and Molecular Engineering, Peking University, Beijing 100871, China

Table of Contents

1. **Supplementary Figures 1-38 and Tables 1-2.**
2. **Supplementary NMR Spectra**
3. **Supplementary Notes 1-5.**
4. **Supplementary References**

1. Supplementary Figures 1-38 and Tables 1-2.

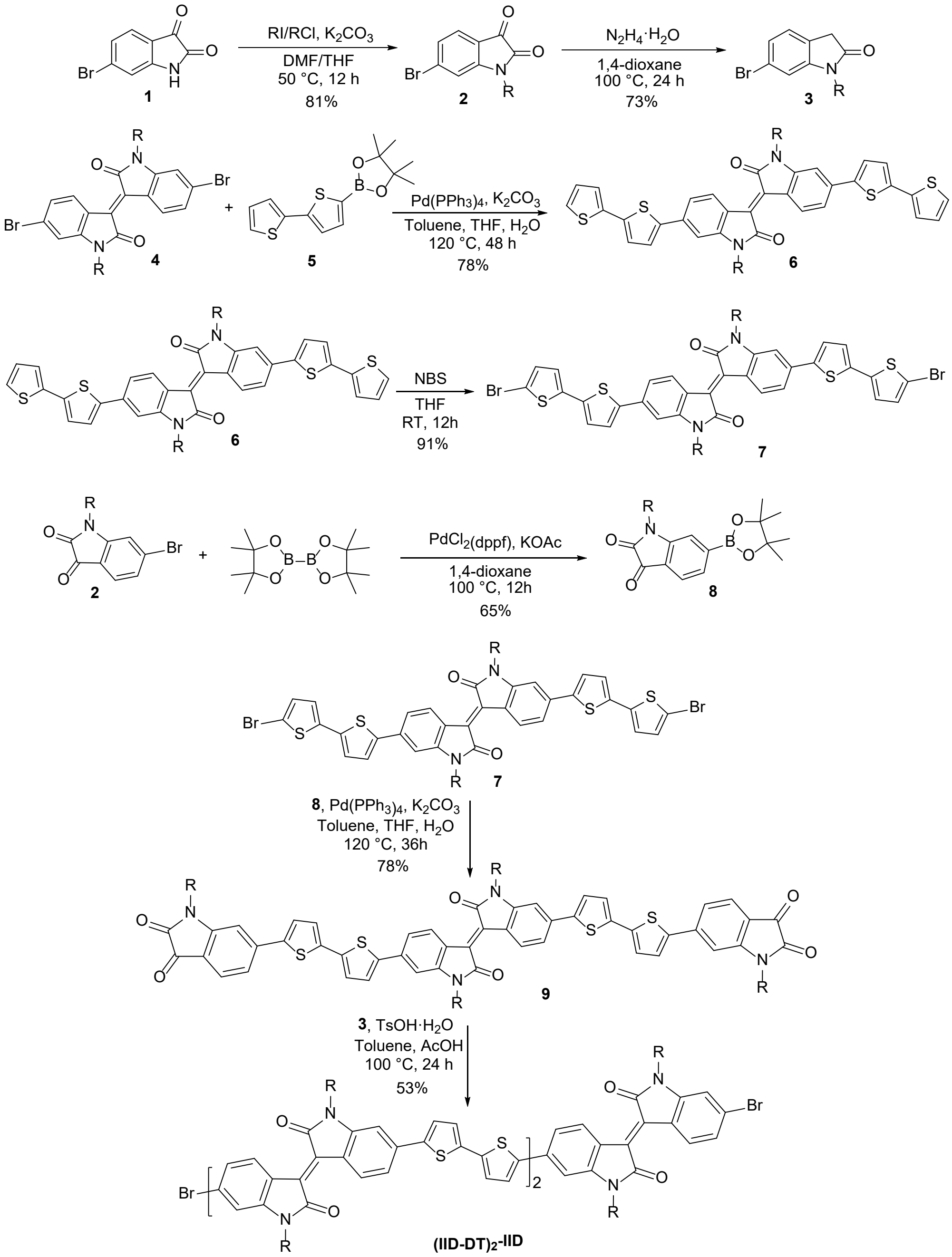

Supplementary Figure 1 | Synthesis of (IID-DT)$_2$-IID.

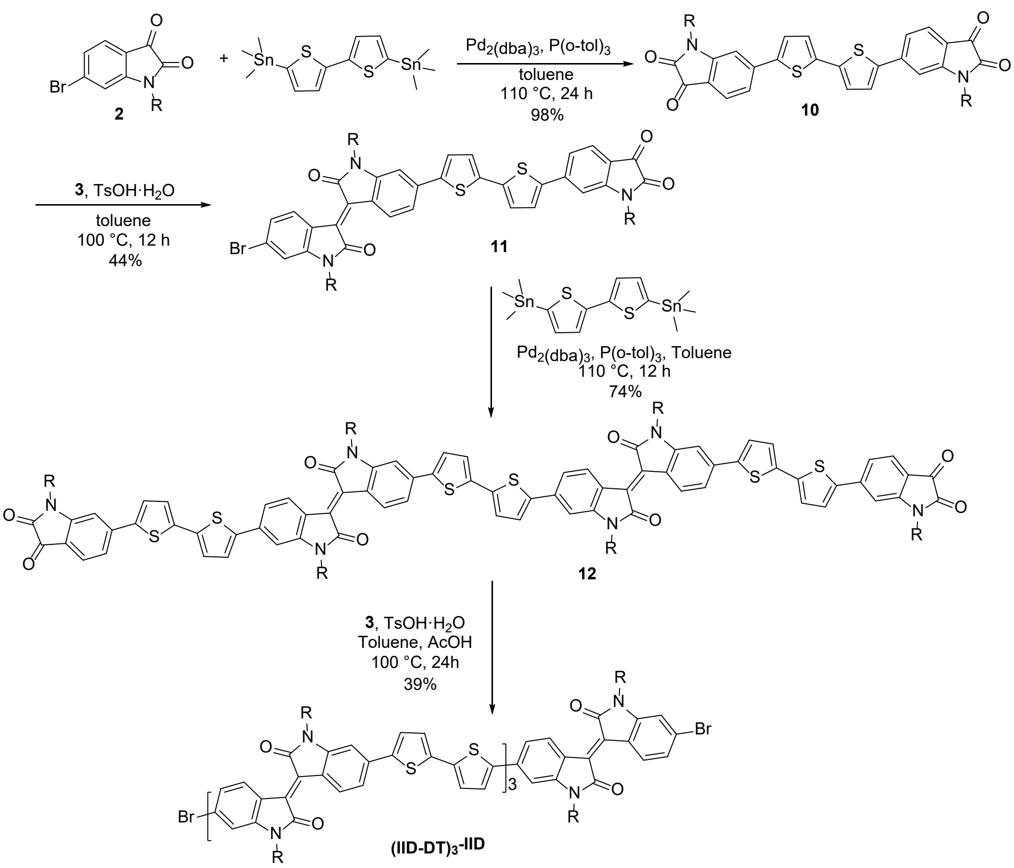

Supplementary Figure 2 | Synthesis of (IID-DT)$_3$-IID.

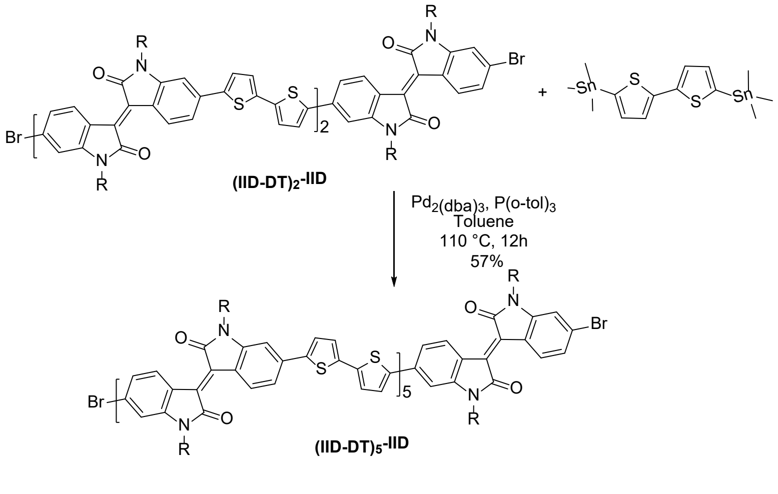

Supplementary Figure 3 | Synthesis of (IID-DT)$_5$-IID.

All commercially available chemicals were used without further purification unless otherwise noted. Compounds **1** and **5** were commercially obtained. Compounds **2** and **3** were synthesized following our reported procedures[1]. The polymers were synthesized following our reported procedures[2].

Synthetic procedures of new compounds

***(E)*-6,6'-dibromo-*N,N'*- (4-decyltetradecyl)-isoindigo *(4)*.** To a solution of 6,6'-dibromoisoindigo (1.70 g, 4.04 mmol), potassium carbonate (1.68 g, 12.1 mmol) in dimethylformamide (DMF) (100 mL), 11-(3-iodopropyl)henicosane (4.33 g, 9.31 mmol) was added under nitrogen. The mixture was stirred for 24 h at 100 ºC and then the solvent was removed under reduced pressure. The residues were purified by silica gel chromatography with eluting (PE:DCM = 5:1) to give **4** as deep-red solids

(4.24 g, 96%).^{1}H NMR (400 MHz, $CDCl_3$, ppm): δ 9.10-9.08 (d, J = 8.6 Hz, 2H), 7.18-7.16 (dd, J = 8.6 Hz, J = 1.8 Hz, 2H), 6.93-6.92 (d, J = 1.7 Hz, 2H), 3.73-3.69 (t, J = 7.4 Hz, 4H), 1.71-1.60 (m, 4H), 1.71-1.60 (m, 4H), 1.32-1.22 (m, 82H), 0.89-0.86 (t, J = 6.8 Hz, 12H). ^{13}C NMR ($CDCl_3$, 101 MHz, ppm): δ 167.7, 145.8, 132.7, 131.2, 126.8, 125.2, 120.5, 111.3, 40.6, 37.1, 33.5, 31.9, 30.8, 30.1, 29.7, 29.7, 29.4, 26.7, 24.5, 22.7, 14.1

(E)-6,6'-di([2,2'-bithiophen]-5-yl)-1,1'-bis(4-decyltetradecyl)-[3,3'-biindolinylidene]-2,2'-dione (6). A mixture of **4** (396 mg, 0.363 mmol), **5** (383 mg, 1.31 mmol), $Pd(PPh_3)_4$ (38 mg, 0.032 mmol), and 4 mL of aqueous potassium carbonate solution (2M) in 15 mL of toluene and 5 mL of THF was added to a Schlenk flask. The flask was charged with N_2 through a freeze-pump-thaw cycle for three times. The mixture was stirred at 120 °C for 48 h. After quenched with water, the mixture was extracted with chloroform, the organic layers were combined and washed with brine, and then dried over anhydrous Na_2SO_4. After the solvent was removed under reduced pressure, the residue was purified by column chromatography on silica gel with eluent (PE:DCM = 2:1) to give **6** as purple solids (358 mg, 78%). ^{1}H NMR (400 MHz, CD_2Cl_2, ppm): δ 9.21-9.19 (d, J = 8.3 Hz, 2H), 7.39-7.38 (d, J = 8.0 Hz, 2H), 7.28 (t, J = 1.4 Hz, 2H), 7.27-7.25 (m, 4H), 7.21-7.20 (d, J = 4.1 Hz, 2H), 7.06-7.04 (dd, J = 5 Hz, J = 3.6 Hz, 2H), 7.00 (d, J = 1.7 Hz, 2H), 3.82-3.78 (t, J = 7.0 Hz, 4H), 1.74-1.68(m, 4H), 1,38-1.20 (m, 78H), 0.85-0.82 (t, J = 6.6 Hz, 12H). ^{13}C NMR ($CDCl_3$, 101 MHz, ppm): δ 168.7, 145.6, 142.9, 138.5, 137.7, 137.5, 132.1, 130.8, 130.7, 128.3, 125.4, 125.2, 125.1, 124.4, 121.5, 119.3, 40.6, 37.4, 33.9, 32.3, 31.6, 30.5, 30.1, 29.7, 29.3, 24.9, 23.4, 14.5. MALDI-HRMS calcd. for $C_{80}H_{115}N_2O_2S_4$ ($[M + H]^+$): 1263.7836; found: 1263.7855.

(E)-6,6'-bis(5'-bromo-[2,2'-bithiophen]-5-yl)-1,1'-bis(4-decyltetradecyl)-[3,3'-biindolinylidene]-2,2'-dione (7). To a solution of **6** (177 mg, 0.135mmol) in anhydrous THF (40 mL) was added a solution of NBS (53 mg, 0.298 mmol) in anhydrous THF (20 mL) slowly at room temperature. The mixture was stirred at room temperature for 12 h. After quenched with water, the mixture was extracted with chloroform. The organic layers were combined and washed with brine, and then dried over anhydrous Na_2SO_4. After the solvent was removed under reduced pressure, the residue was purified by column chromatography on silica gel with eluent (PE:CH_2Cl_2 = 3:1) to give **7** as purple solids (191 mg, 91%). ^{1}H NMR (400 MHz, CD_2Cl_2, ppm): δ 9.20-9.18 (d, J = 8.4 Hz, 2H), 7.35-7.36 (d, J = 3.8 Hz, 2H), 7.25-7.23 (dd, J = 8.4 Hz, J = 1.7 Hz, 2H), 7.13-7.12 (d, J = 3.8 Hz, 2H), 7.01-6.98 (m, 4H), 6.95 (d, J = 1.6 Hz, 2H), 3.81-3.77 (t, J = 6.9 Hz, 4H), 1.75-1.68 (m, 4H), 1.39-1.36 (m, 6H), 1.25-1.20 (m, 72H), 0.86-0.82 (t, J = 6.7 Hz, 12H). ^{13}C NMR ($CDCl_3$, 101 MHz, ppm): δ 168.4, 145.5, 143.3, 138.9, 137.4, 137.2, 132.0, 131.1, 130.8, 125.3, 125.2, 124.3, 121.6, 119.2, 111.8, 104.5, 40.8, 37.4, 33.9, 32.3, 31.2, 30.5, 30.1, 30.0, 29.7, 27.1, 24.9, 23.1, 14.5. MALDI-HRMS calcd. for $C_{80}H_{113}Br_2N_2O_2S_4$ ($[M + H]^+$): 1419.6046; Found: 1419.6068.

1-(4-decyltetradecyl)-6-(4,4,5,5-tetramethyl-1,3,2-dioxaborolan-2-yl)indoline-2,3-dione (8). A mixture of **2** (283 mg, 0.504 mmol), bis(pinacolato)diboron (282 mg, 1.109 mmol), anhydrous KOAc (148 mg, 1.512 mmol), $PdCl_2$(dppf) (36 mg, 0.050 mmol) in anhydrous 1,4-dioxane (8 mL) was added to a Schlenk flask under N_2. The mixture was stirred at 100 °C for 12 h. After quenched with water, the mixture was extracted with DCM, the organic layers were combined and then washed with brine, and then dried over anhydrous Na_2SO_4. After the solvent was removed under reduced pressure, the residue was purified by column chromatography on silica gel with eluent (PE:EtOAc = 2:1) to give **8** as orange oil (200mg, 65%). ^{1}H NMR (400 MHz, CD_2Cl_2, ppm): δ 7.54-7.49 (m, 2H), 7.25 (s, 1H), 3.70-3.66 (t, J = 7.2 Hz, 2H), 1.70-1.64 (m, 2H), 1.33-1.28 (m, 12H), 1.23-1.87 (m, 41H), 0.86-0.84 (t, J = 6.7 Hz, 6H). ^{13}C NMR (CD_2Cl_2, 101 MHz, ppm): δ 186.0, 158.6, 150.7,

130.4, 124.4, 119.9, 115.9, 85.2, 78.1, 41.1, 37.6, 34.0, 32.5, 31.3, 30.7, 30.3, 29.9, 27.1, 25.2, 24.9, 23.3, 14.5. MALDI-HRMS calcd. for $C_{38}H_{65}BNNaO_4$ ([M + Na]$^+$): 632.4821; Found: 632.4839.

(E)-6,6'-((1,1'-bis(4-decyltetradecyl)-2,2'-dioxo-[3,3'-biindolinylidene]-6,6'-diyl)bis([2,2'-bithiophene]-5',5-diyl))bis(1-(4-decyltetradecyl)indoline-2,3-dione) (9). A mixture of **7** (277 mg, 0.194 mmol), **8** (297 mg, 0.487 mmol), $Pd(PPh_3)_4$ (15 mg, 0.012 mmol), and 4 mL of aqueous potassium carbonate solution (2M) in 15 mL of toluene and 5 mL of THF was added to a Schlenk flask. The flask was charged with N_2 through a freeze-pump-thaw cycle for three times. The mixture was refluxed for 16 h. After quenched with water, the mixture was extracted with chloroform, the organic layers were combined and washed with brine, and then dried over anhydrous Na_2SO_4. After the solvent was removed under reduced pressure, the residue was purified by column chromatography on silica gel with eluent (DCM:EtOAc = 25:1) to give **9** as black solids (358 mg, 78%). ^{1}H NMR (400 MHz, CD_2Cl_2, ppm): δ 9.22-9.20 (d, J = 8.3 Hz, 2H), 7.58-7.56 (d, J = 8.0 Hz, 2H), 7.47-7.46 (d, J = 4.0 Hz, 2H), 7.41-7.40 (d, J = 4.0 Hz, 2H), 7.33-7.31 (dd, J = 7.8 Hz, J = 1.4 Hz, 2H), 7.27-7.26 (m, 6H), 7.05 (s, 2H), 6.97 (s, 2H), 3.83-3.80 (t, J = 6.3 Hz, 4H), 3.74-3.71 (t, J = 6.8 Hz, 4H), 1.72 (m, 8H), 1.58 (m, 10H), 1.24-1.20 (m, 146H), 0.84-0.80 (24H). ^{13}C NMR ($CDCl_3$, 101 MHz, ppm): δ 182.2, 174.2, 168.1, 158.7, 151.8, 147.3, 145.1, 143.9, 143.4, 141.1, 137.3, 131.5, 127.3, 125.9, 125.5, 121.2, 120.5, 119.1, 118.1, 117.2, 116.1, 108.9, 106.3, 104.2, 37.5, 33.6, 33.0, 31.4, 30.9, 30.2, 29.8, 29.7, 29.4, 26.6, 24.3, 22.0, 14.1. MALDI-HRMS calcd. for $C_{144}H_{217}N_4O_6S_4$ ([M + H]$^+$): 2226.5676; Found: 2226.5740.

(IID-DT)$_2$-IID. To a solution of **9** (250 mg, 0.112 mmol) and $TsOH{\cdot}H_2O$ (21 mg, 0.112 mmol) in toluene (20 mL) and HOAc (6 mL), a solution of **3** (135 mg, 0.247 mmol) in toluene (10 mL) was added. The mixture was refluxed for 24 h. After quenched with water, the mixture was extracted with chloroform, the organic layers were combined and washed with brine, and then dried over anhydrous Na_2SO_4. After the solvent was removed under reduced pressure, the residue was purified by column chromatography on silica gel with eluent (PE:DCM = 2:1) to give a crude product. The crude product was purified with preparative GPC with $CHCl_3$ as the eluent to give **(IID-DT)$_2$-IID** as black solids (195 mg, 53%). ^{1}H NMR (400 MHz, CD_2Cl_2, ppm): δ 8.92-8.88 (m, 6H), 7.03-7.01 (m, 4H), 6.89-6.80 (m, 6H), 6.75 (m, 4H), 6.64 (m, 4H), 6.43-6.40 (m, 4H), 3.67-3.58 (m, 12H), 1.71-1.59 (m, 12H), 1.44-1.25 (m, 234H), 0.87-0.83 (m, 36H). ^{13}C NMR (101 MHz, $CDCl_3$): δ 168.0, 167.9, 167.6, 162.5, 153.8, 149.3, 145.3, 145.2, 144.8, 142.9, 142.5, 137.9, 137.4, 137.1, 136.4, 132.6, 131.0, 130.7, 125.9, 124.6, 121.1, 120.8, 120.6, 110.9, 103.9, 103.5, 97.1, 37.2, 33.6, 32.0, 31.0, 30.3, 30.2, 29.8, 29.8, 29.7, 29.4, 26.7, 22.7, 14.2. MALDI-HRMS calcd. for $C_{208}H_{321}Br_2N_6O_6S_4$ ([M + H]$^+$): 3285.2242; Found: 3285.2244.

6,6'-([2,2'-bithiophene]-5,5'-diyl)bis(1-(4-decyltetradecyl)indoline-2,3-dione) (10). A mixture of **2** (200 mg, 0.355 mmol), 2,5-bis (trimethylstannyl)thiophene (83 mg, 0.169 mmol), $Pd_2(dba)_3$ (6.2 mg, 4 mol%), P(*o*-tol)$_3$ (8.2 mg, 16 mol%) in 10 mL of toluene was added to a Schlenk flask. The flask was charged with N_2 through a freeze-pump-thaw cycle for three times. The mixture was refluxed for 24 h. After quenched with water, the mixture was extracted with DCM, the organic layers were combined and washed with brine, and then dried over anhydrous Na_2SO_4. After the solvent was removed under reduced pressure, the residue was purified by column chromatography on silica gel with eluent (DCM:EtOAc = 100:1) to give **10** as red solids (187 mg, 98%). ^{1}H NMR (400 MHz, $CDCl_3$, ppm): δ 7.64-7.62 (d, J = 7.8 Hz, 2H), 7.47-7.46 (d, J = 3.9 Hz, 2H), 7.35-7.33 (dd, J = 7.9 Hz, J = 1.3 Hz, 2H), 7.31-7.30 (d, J = 4.0 Hz, 2H), 7.04 (s, 2H), 3.78-3.74 (t, J = 7.4 Hz, 4H), 1.76-1.69 (m, 4H), 1.36-1.35 (m, 2H), 1.27-1.22 (m, 72H), 0.87-0.84 (t, J = 6.7 Hz, 12H). ^{13}C NMR

($CDCl_3$, 101 MHz, ppm): δ 182.4, 158.9, 152.1, 143.4, 142.30, 139.2, 127.3, 126.5, 126.1, 120.6, 116.8, 106.6, 40.9, 37.3, 33.8, 32.2, 30.9, 30.4, 30.0, 29.9, 29.7, 26.9, 24.7, 23.0, 14.4. MALDI-HRMS calcd. for $C_{72}H_{109}N_2O_4S_2$ ($[M + H]^+$): 1129.7723; Found: 1129.7783.

(E)-6-bromo-1,1'-bis(4-decyltetradecyl)-6'-(5'-(1-(4-decyltetradecyl)-2,3-dioxoindolin-6-yl)-[2,2'-bithiophen]-5-yl)-[3,3'-biindolinylidene]-2,2'-dione (11). To a solution of **10** (154 mg, 0.136 mmol) and $TsOH{\bullet}H_2O$ (21 mg, 0.109 mmol) in toluene (30 mL), a solution of **3** (75 mg, 0.136 mmol) in toluene (10 mL) was added dropwise. The mixture was refluxed for 12 h. After quenched with water, the mixture was extracted with DCM, the organic layers were combined and washed with brine, and then dried over anhydrous Na_2SO_4. After the solvent was removed under reduced pressure, the residue was purified by column chromatography on silica gel with eluent (DCM:PE = 2:1) to give **11** as purple solids (100 mg, 44%). ^{1}H NMR (400 MHz, $CDCl_3$, ppm): δ 9.21-9.19 (d, J = 8.4 Hz, 1H), 9.09-9.07 (d, J =8.6 Hz, 1H), 7.57-7.55 (d, J = 7.9 Hz, 1H), 7.45-7.44 (d, J = 4.0 Hz, 1H), 7.40-7.39 (d, J = 3.9 Hz, 1H), 7.31-7.29 (dd, J = 7.9 Hz, J = 1.2 Hz, 1H), 7.26-7.24 (m, 3H), 7.14-7.12 (dd, J = 8.6 Hz, J = 1.8 Hz, 1H), 7.03 (s, 1H), 6.95-6.93 (dd, J = 5.0 Hz, J = 1.6 Hz, 2H), 3.79-3.75 (t, J = 7.3 Hz, 2H), 3.74-3.66 (m, 4H), 1.70-1.65 (m, 6H), 1.24-1.21 (m, 117H), 0.96-0.82 (m, 18H). ^{13}C NMR ($CDCl_3$, 101 MHz, ppm): δ 182.30, 168.24, 167.94, 158.96, 151.96, 145.71, 145.61, 143.91, 143.43, 141.44, 139.95, 137.39, 137.28, 132.93, 131.50, 131.37, 131.04, 127.19, 126.59, 126.30, 125.79, 125.50, 125.31, 125.15, 121.48, 120.83, 120.26, 119.17, 116.54, 111.35, 106.29, 104.37, 40.86, 40.73, 37.43, 37.35, 33.84, 33.78, 32.24, 31.16, 31.01, 30.48, 30.46, 30.06, 30.04, 29.69, 27.01, 24.85, 24.78, 23.01, 14.44. MALDI-HRMS calcd. for $C_{104}H_{161}BrN_3O_4S_2$ ($[M + H]^+$): 1659.1106; Found: 1659.1136.

(3E,3''E)-6',6'''-([2,2'-bithiophene]-5,5'-diyl)bis(1,1'-bis(4-decyltetradecyl)-6-(5'-(1-(4-decyltetradecyl)-2,3-dioxoindolin-6-yl)-[2,2'-bithiophen]-5-yl)-[3,3'-biindolinylidene]-2,2'-dione) (12). A mixture of **11** (100 mg, 0.060 mmol), 2,5-bis (trimethylstannyl)thiophene (14.1 mg, 0.028 mmol), $Pd_2(dba)_3$ (1.1 mg, 4 mol%), and P(*o*-tol)$_3$ (1.4 mg, 16 mol%) in 10 mL of toluene was added to a Schlenk flask. The flask was charged with N_2 through a freeze-pump-thaw cycle for three times. The mixture was refluxed for 12 h. After quenched with water, the mixture was extracted with chloroform, the organic layers were combined and washed with brine, and then dried over anhydrous Na_2SO_4. After the solvent was removed under reduced pressure, the residue was purified by column chromatography on silica gel with eluent (DCM:EtOAc = 20:1) to give **12** as black solids (74 mg, 74%). ^{1}H NMR (400 MHz, $CDCl_3$, ppm): δ 8.89-8.84 (m, 4H), 7.37-7.35 (d, J = 7.6 Hz, 2H), 7.16-7.15 (d, J = 3.6 Hz, 2H), 7.05-7.03 (d, J = 7.6 Hz, 2H), 7.00-6.97 (m, 4H), 6.81-6.75 (m, 10H), 6.61 (s, 2H), 6.40 (s, 2H), 6.33 (s, 2H), 3.67-3.62 (m, 12H), 1.74-1.62 (m, 12H), 1.34-1.24 (m, 234H), 0.87-0.83 (m, 36H). ^{13}C NMR ($CDCl_3$, 101 MHz, ppm): δ 182.2, 168.3, 168.3, 158.9, 151.8, 145.0, 144.9, 143.9, 143.3, 143.3, 142.8, 141.3, 134.0, 137.7, 136.7, 136.7, 136.2, 136.1, 131.5, 130.9, 130.9, 130.7, 130.7, 126.9, 126.1, 125.5, 124.9, 124.7, 121.6, 121.2, 120.1, 116.5, 106.3, 103.8, 88.4, 40.7, 40.7, 37.6, 37.4, 33.9, 33.8, 32.3, 31.5, 31.1, 30.6, 30.5, 30.2, 30.1, 29.7, 27.1, 27.0, 25.1, 24.9, 23.0, 14.5. MALDI-HRMS calcd. for $C_{216}H_{325}N_6O_8S_6$ ($[M + H]^+$): 3323.3528; Found: 3323.3499.

(IID-DT)$_3$-IID. To a solution of **12** (74 mg, 0.020 mmol) and $TsOH{\cdot}H_2O$ (6.8 mg, 0.017 mmol) in toluene (10 mL) and HOAc (5 mL), a solution of **3** (28 mg, 0.051 mmol) in toluene (10 mL) was added. The mixture was refluxed for 24 h. After quenched with water, the mixture was extracted with chloroform, the organic layers were combined and washed with brine, and then dried over anhydrous Na_2SO_4. After the solvent was removed under reduced pressure, the residue was purified by column chromatography on silica gel with eluent (PE:DCM = 1:1) to give a crude product. The

crude product was purified with preparative GPC with $CHCl_3$ as the eluent to give **(IID-DT)$_3$-IID** as black solids (38 mg, 39%). ^{1}H NMR (500 MHz, $C_2Cl_4D_2$, 90 °C, ppm): δ 9.24-9.23 (m, 6H), 9.13-9.11 (d, J = 8.6 Hz, 2H), 7.37-7.36 (m, 6H), 7.30-7.27 (m, 6H), 7.24-7.23 (m, 6H), 7.19-7.18 (dd, J = 8.6 Hz, J = 1.6 Hz, 2H), 6.96 (m, 6H), 6.94 (m, 2H), 3.87-3.74 (m, 16H), 1.82-1.74 (m, 16H), 1.43-1.29 (m, 312H), 0.93-0.89 (m, 48H). ^{13}C NMR (125 MHz, $C_2Cl_4D_2$, 90 °C, ppm): δ 168.0, 167.5, 145.8, 145.3, 143.1, 142.8, 138.3, 137.6, 137.0, 133.6, 132.9, 131.7, 131.5, 131.1, 131.0, 130.5, 129.9, 128.9, 126.0, 125.0, 124.6, 123.2, 121.5, 120.7, 120.3, 119.2, 118.8, 112.1, 111.0, 104.1, 103.9, 40.5, 37.8, 34.2, 31.7, 31.2, 30.0, 29.5, 29.1, 26.7, 24.7, 22.4, 13.8. MALDI-HRMS calcd. for $C_{280}H_{429}Br_2N_8O_8S_6$ ([M + H]$^+$): 4382.0094; Found: 4381.9937.

(IID-DT)$_5$-IID. **2** (167 mg, 0.051 mmol), 2,5-bis (trimethylstannyl)thiophene (5.86 mg, 0.012 mmol), $Pd_2(dba)_3$ (0.46 mg, 4 mol%), P(o-tol)$_3$ (0.61 mg, 16 mol%) and 10 mL of toluene were added to a Schlenk flask. The flask was charged with N_2 through a freeze-pump-thaw cycle for three times. The mixture was refluxed for 12 h. The crude product was purified with preparative GPC with $CHCl_3$ as the eluent to give **(IID-DT)$_5$-IID** as black solids (45 mg, 57%). ^{1}H NMR (500 MHz, $C_2Cl_4D_2$, 90 °C, ppm): δ 9.24-9.09 (m, 12H), 7.40-6.84 (m, 50H), 3.86-3.77 (m, 24H), 1.81-1.78 (m, 24H), 1.31 (m, 468H), 0.93-0.91 (m, 72H). MALDI-HRMS calcd. for $C_{424}H_{645}Br_2N_{12}O_{12}S_{10}$ ([M + H]$^+$): 6575.5798; Found: 6575.5775.

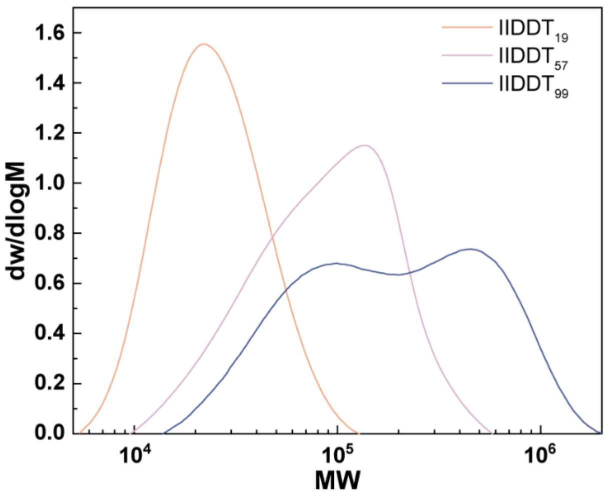

Supplementary Figure 4 | Molecular weights and dispersity of the polymers. The molecular weight distributions of these polymers were measured by high-temperature gel permeation chromatography (GPC).

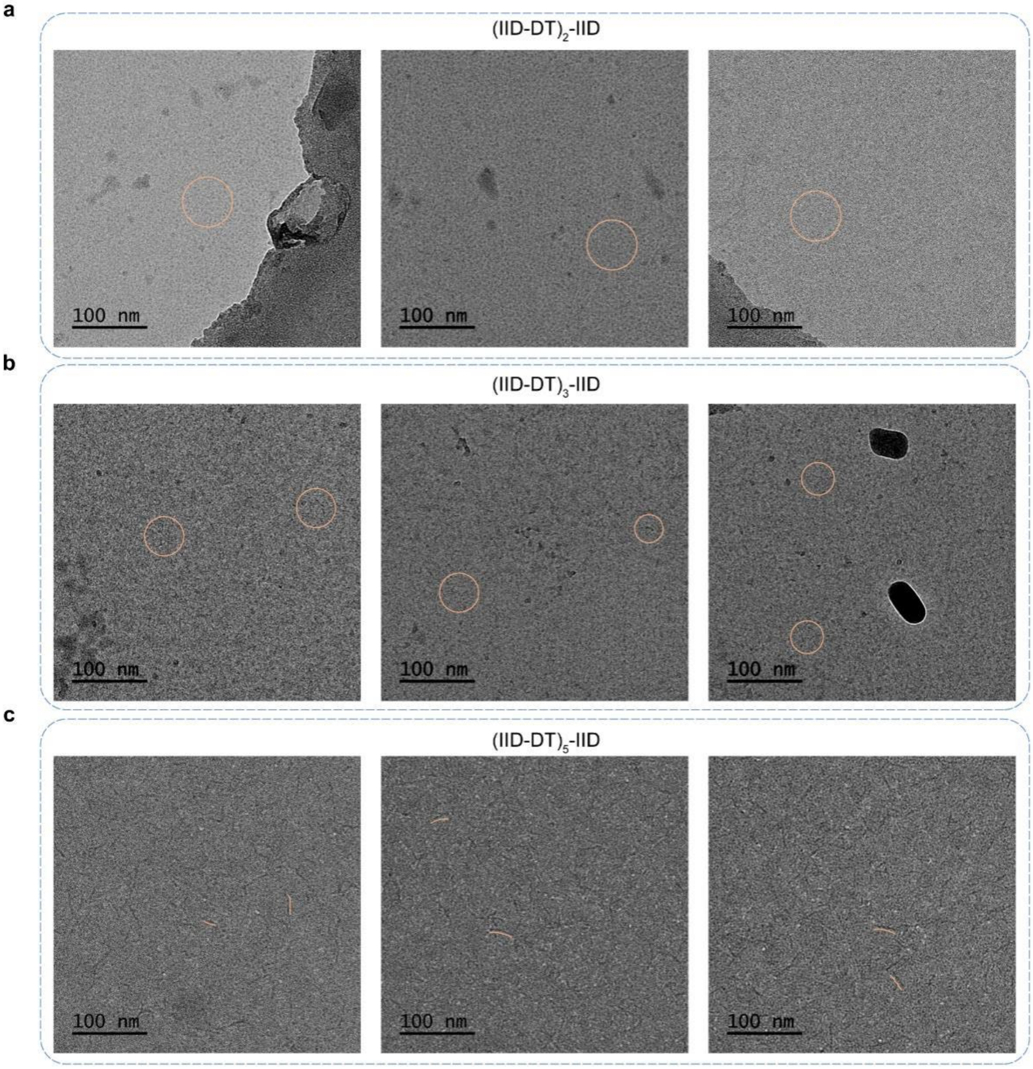

Supplementary Figure 5 | Cryo-EM images of 0.1 g·L^{-1} oligomer solutions. (a) (IID-DT)$_2$-IID, **(b)** (IID-DT)$_3$-IID, and **(c)** (IID-DT)$_5$-IID. Every oligomer sample shows three images. The samples of (IID-DT)$_2$-IID show spot-like shadows with diameter of 4-5 nm, which are consistent of the calculated molecular size. The samples of (IID-DT)$_3$-IID show short fibers with 7-8 nm long. The samples of (IID-DT)$_3$-IID show longer fibers with 17-32 nm long. The image contrasts of (IID-DT)$_2$-IID and (IID-DT)$_3$-IID are both limited by small molecular sizes.

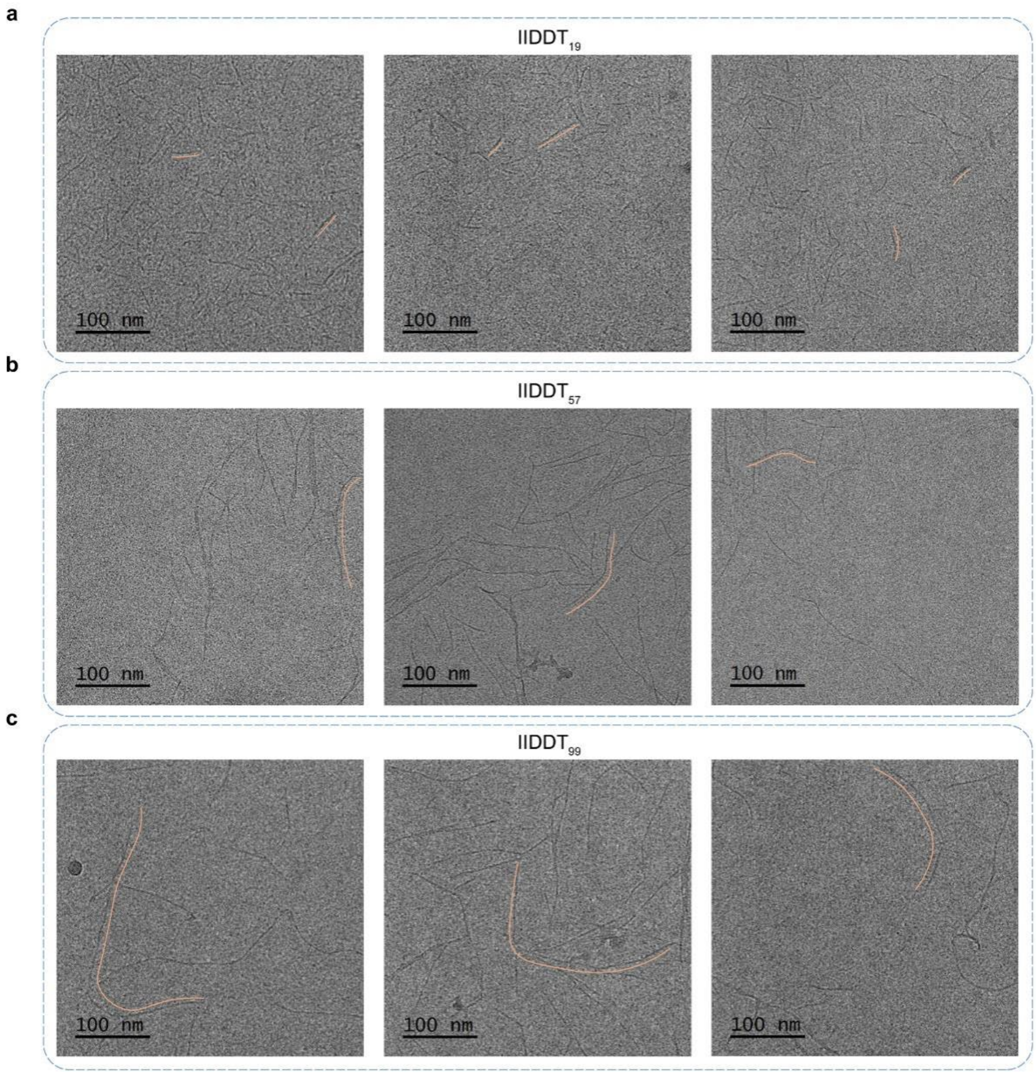

Supplementary Figure 6 | Cryo-EM images of 0.1 g·L^{-1} polymer solutions. (a) IIDDT$_{19}$, **(b)** IIDDT$_{57}$, and **(c)** IIDDT$_{99}$. Every polymer sample shows three images.

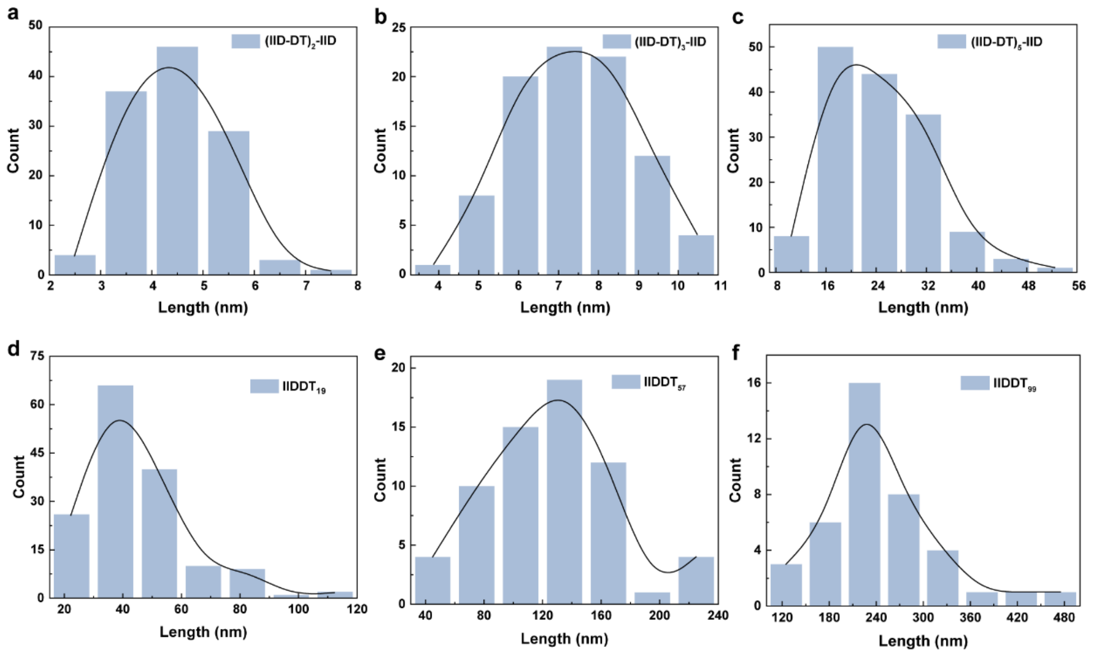

Supplementary Figure 7 | Histograms and distribution of fiber lengths for the samples. (a) (IID-DT)$_2$-IID, **(b)** (IID-DT)$_3$-IID, **(c)** (IID-DT)$_5$-IID, **(d)** IIDDT$_{19}$, **(e)** IIDDT$_{57}$, and **(f)** IIDDT$_{99}$. These data were counted from above cryo-EM images. The distribution of (IID-DT)$_2$-IID were collected from 120 data; The distribution of (IID-DT)$_3$-IID were collected from 90 data; The distribution of (IID-DT)$_5$-IID were collected from 150 data; The distribution of IIDDT$_{19}$ were collected from 154 data; The distribution of IIDDT$_{57}$ were collected from 65 data; The distribution of IIDDT$_{99}$ were collected from 40 data.

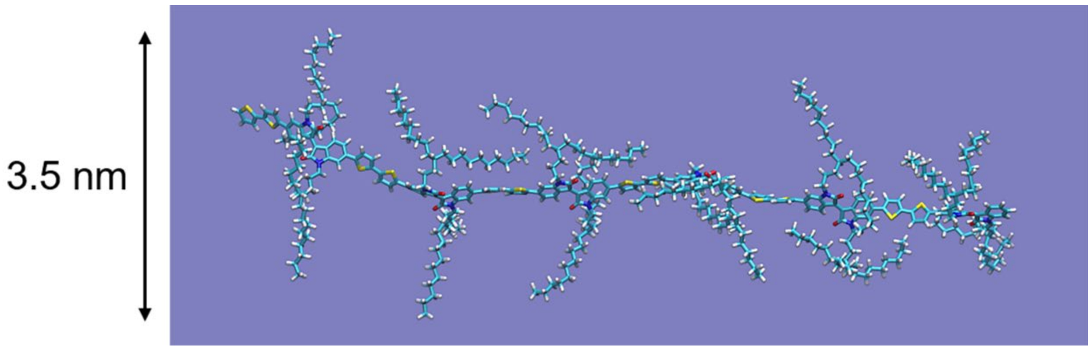

Supplementary Figure 8 | Simulated molecular conformation. The simulated molecular conformation of a single chain with 6 repeating units.

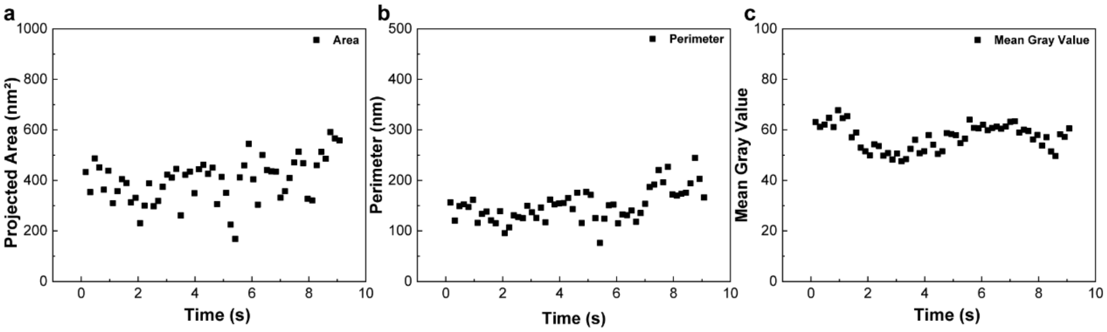

Supplementary Figure 9 | Imaging reliability of Supplementary Movie 1. (a) Projected size, **(b)** perimeter and **(c)** mean gray value versus time of Supplementary Movie 1. The increase indicates the disaggregation process.

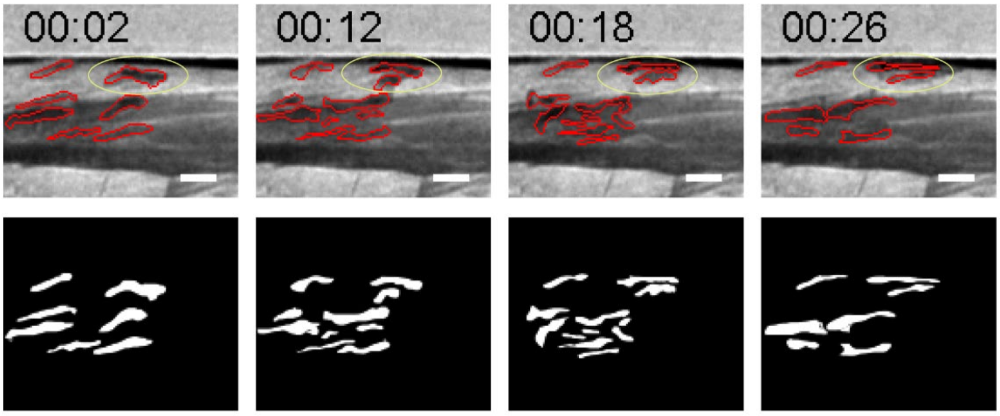

Supplementary Figure 10 | Dynamic disaggregation process of IIDDT$_{19}$. The EM images extracted from the dynamic disaggregation process of IIDDT$_{19}$ in a liquid pocket (extracted from Supplementary Movie 2). The yellow circles mark the typical aggregate which showed disaggregation process. Scale bar: 20 nm. Imaging condition: 80 keV, 9.6 $e^{-1}\cdot Å^{-2}\cdot s^{-1}$.

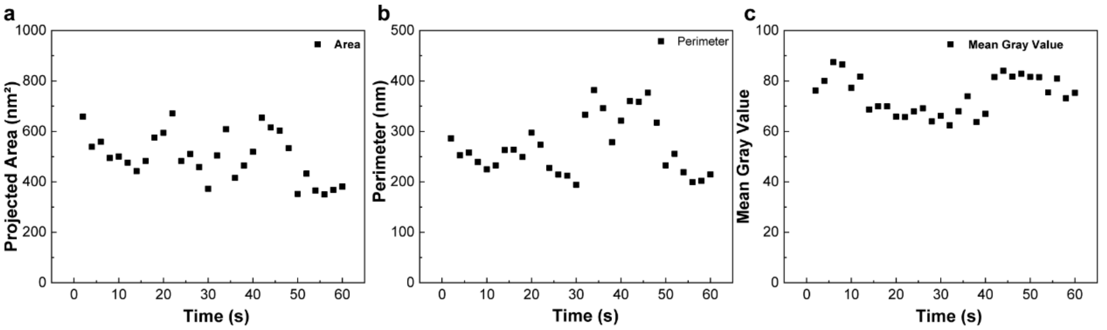

Supplementary Figure 11 | Imaging reliability of Supplementary Movie 2. (a) Projected size, **(b)** perimeter and **(c)** mean gray value versus time of Supplementary Movie 2. The increase indicates the disaggregation process.

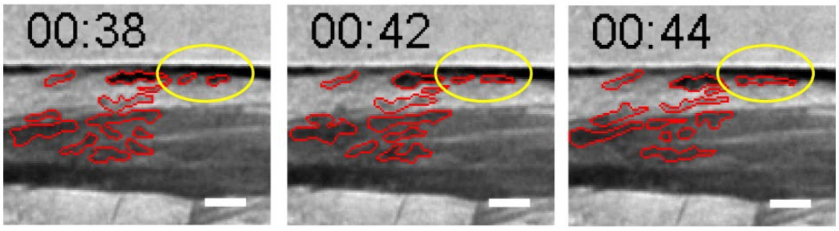

Supplementary Figure 12 | Reaggregation process of polymer strands. Extracted images from Supplementary Movie 2, which exhibited a reaggregate process of polymer strands. Scale bar: 20 nm. The yellow circles mark the typical aggregate which showed reaggregation process.

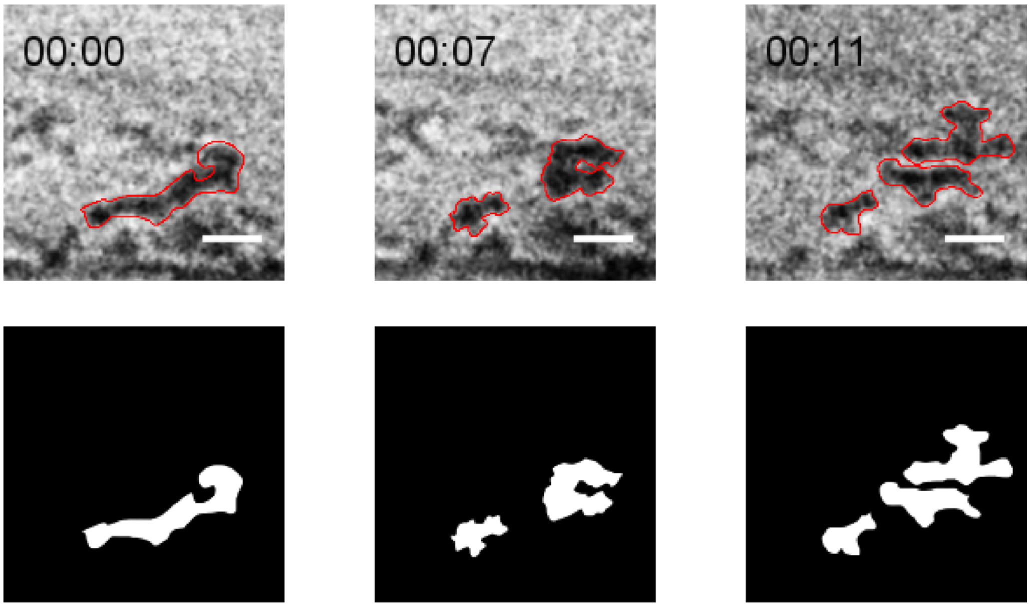

Supplementary Figure 13 | Dynamic disaggregation process of (IID-DT)$_5$-IID. The EM images extracted from the dynamic disaggregation process of (IID-DT)$_5$-IID in a liquid pocket. Scale bar: 20 nm. Imaging condition: 80 keV, 3.9 $e^{-1}\cdot Å^{-2}\cdot s^{-1}$. Rolling average of consecutive five frames was applied to the image series to average out noises in the background.

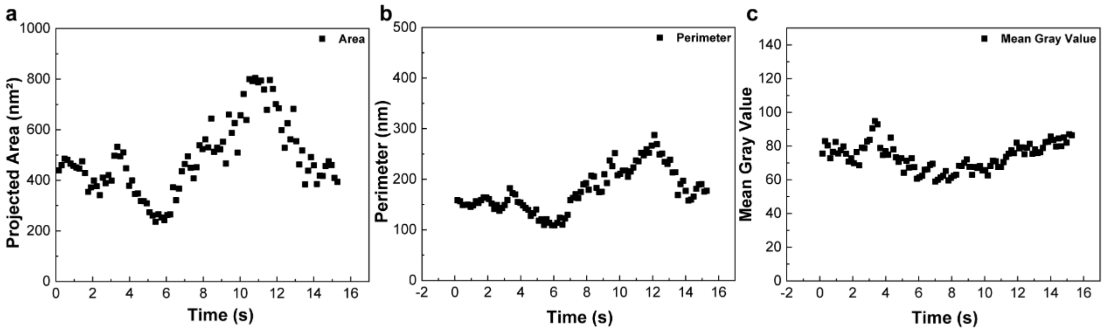

Supplementary Figure 14 | Imaging reliability of Supplementary Movie 3. (a) Projected size, **(b)** perimeter and **(c)** mean gray value versus time of Supplementary Movie 3. The increase indicates the disaggregation process.

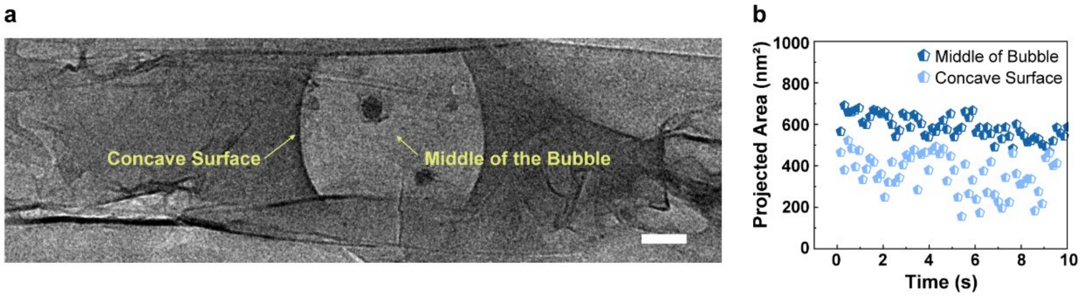

Supplementary Figure 15 | Relationship between the location of aggregates and the disassemble process. (a) Image of a bubble in the liquid cell. The location of concave surface and middle of the bubble were marked by arrows. Scale bar: 50 nm. **(b)** Projected sizes versus time of the aggregate at middle of bubble and concave surface.

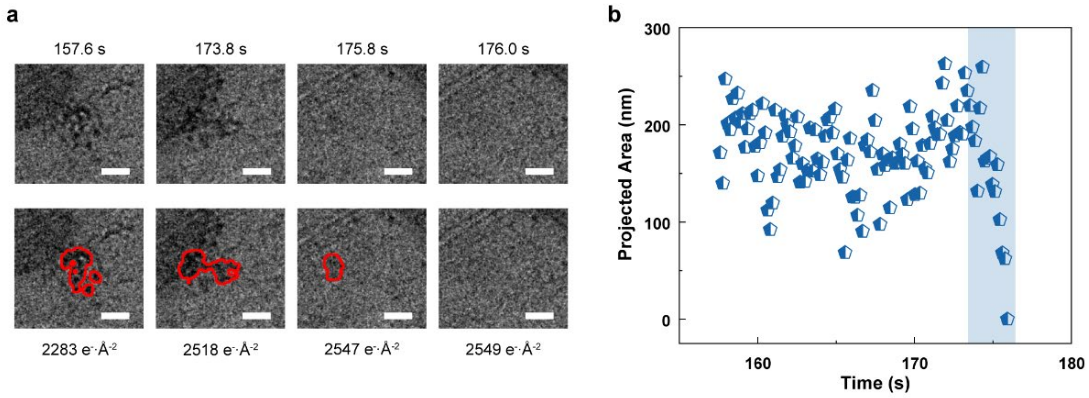

Supplementary Figure 16 | Electron dose of LP-EM. (a) Representative images of the damage process of polymer strands under continuous electron beam irradiation. Scale bar: 20 nm. **(b)** Projected size versus time of this process. Our polymer can withstand at least 1000 $e^{-}\cdot Å^{-2}$. We assessed the total electron dose of Supplementary Movies 1, 2, and 3, which were 116 $e^{-}\cdot Å^{-2}$, 557 $e^{-}\cdot Å^{-2}$, and 59 $e^{-}\cdot Å^{-2}$, respectively, and concluded that all analyzed polymer strands remained intact.

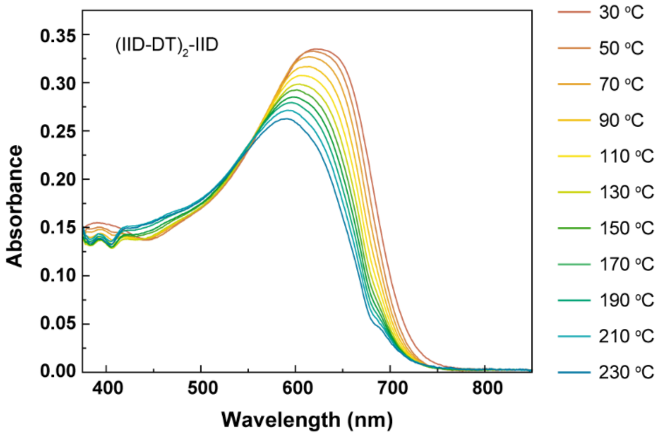

Supplementary Figure 17 | Temperature-dependent absorption spectra. 0.01 $g\cdot L^{-1}$ of (IID-DT)$_2$-IID in 1-chloronaphthalene (CN).

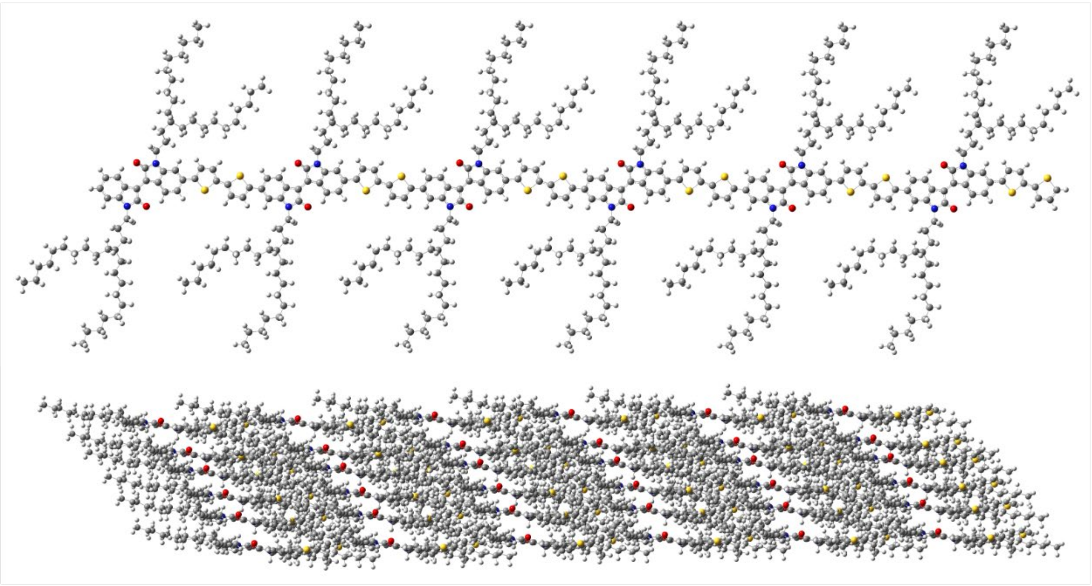

Supplementary Figure 18 | Initial configuration in molecular dynamics. Illustration of the initial models used for the molecular dynamics simulations. Top: A single polymer chain of $(\text{IID-DT})_5$-IID. Bottom: A polymer aggregate made of 6 polymer chains perfectly packed on top of each other.

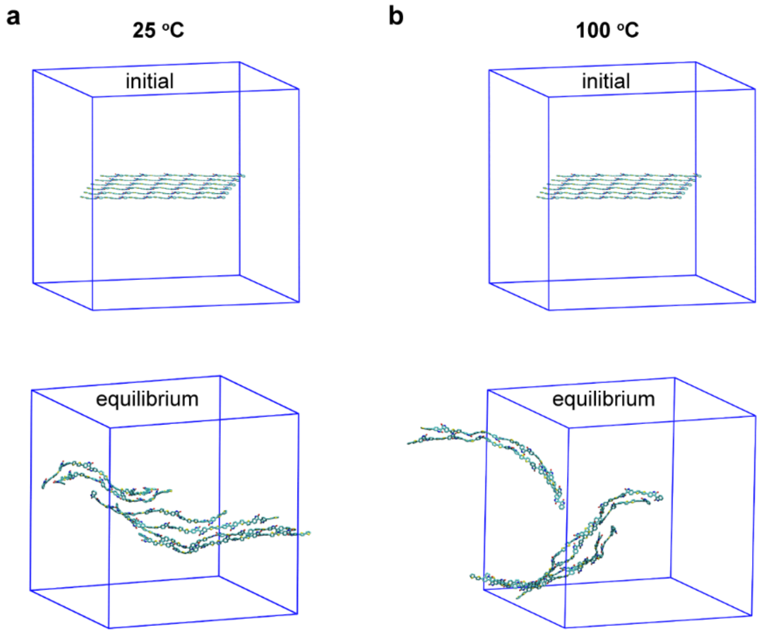

Supplementary Figure 19 | Molecular dynamics simulations of the disaggregated process. (a) Initial model parallel π-π stacked polymer chains (top) and equilibrium snapshot taken from the molecular dynamics simulations at 25 °C. **(b)** Initial model parallel π-π stacked polymer chains (top) and equilibrium snapshot taken from the molecular dynamics simulations at 100 °C.

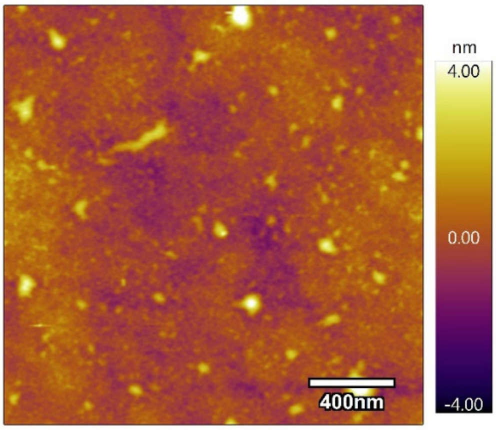

Supplementary Figure 20 | AFM height images of (IID-DT)$_5$-IID freeze-dried from 1 g·L^{-1} *o*DCB solution. As shown in supplementary **Fig.24**, (IID-DT)$_2$-IID and (IID-DT)$_3$-IID didn't aggregate in solutions, because the *UV-vis* absorption spectra of the two oligomer solutions didn't show raised signal at around 710 nm, which was related to the aggregation between interchains. Due to the limitation of resolution ratio, it was difficult to observe discrete molecules of (IID-DT)$_2$-IID and (IID-DT)$_3$-IID samples through AFM.

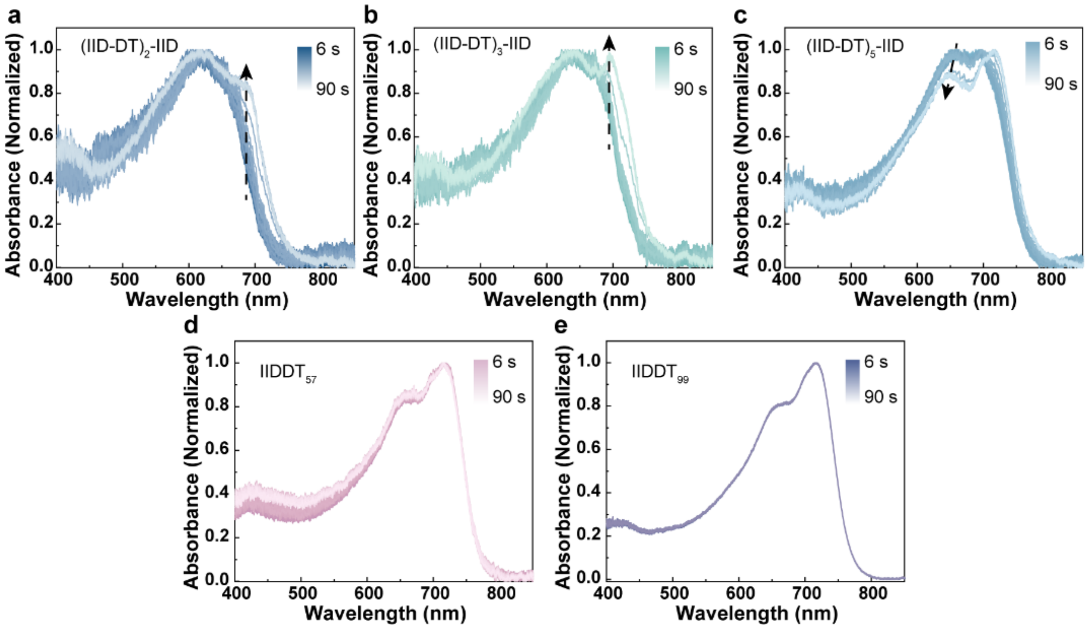

Supplementary Figure 21 | *In situ* normalized UV-*vis* absorption spectra of the film formation process. (a) (IID-DT)$_2$-IID, **(b)** (IID-DT)$_3$-IID, **(c)** (IID-DT)$_5$-IID, **(d)** IIDDT$_{57}$, and **(e)** IIDDT$_{99}$. The dotted arrows marked the trend of signal changes.

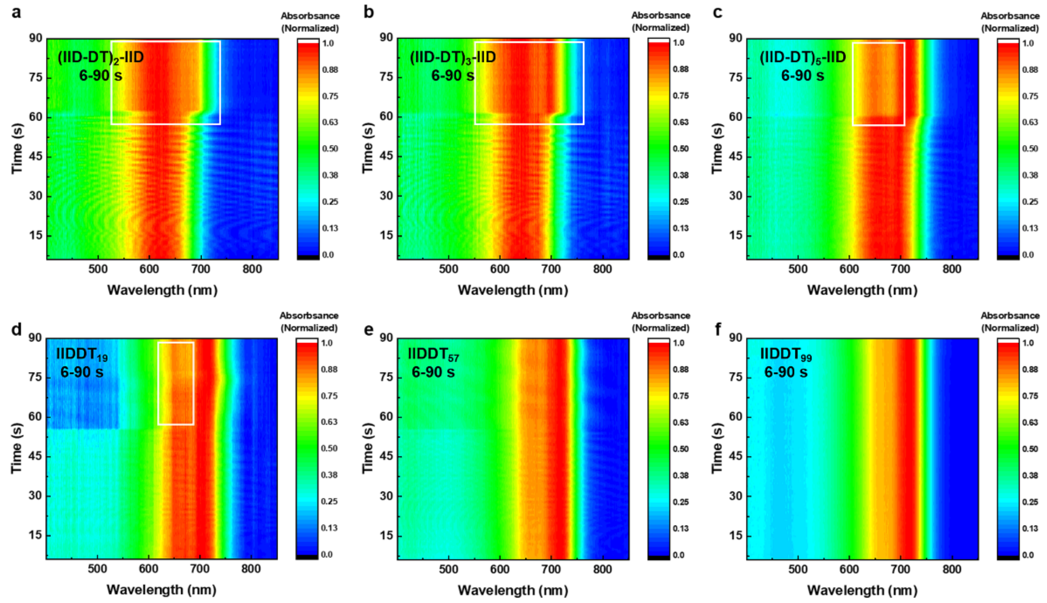

Supplementary Figure 22 | *In situ* normalized UV-*vis* absorption spectra of the film formation process. (a) $(IID\text{-}DT)_2$-IID, **(b)** $(IID\text{-}DT)_3$-IID, **(c)** $(IID\text{-}DT)_5$-IID, **(d)** $IIDDT_{19}$, **(e)** $IIDDT_{57}$, and **(f)** $IIDDT_{99}$. The regions of changed absorbance were marked by white rectangles.

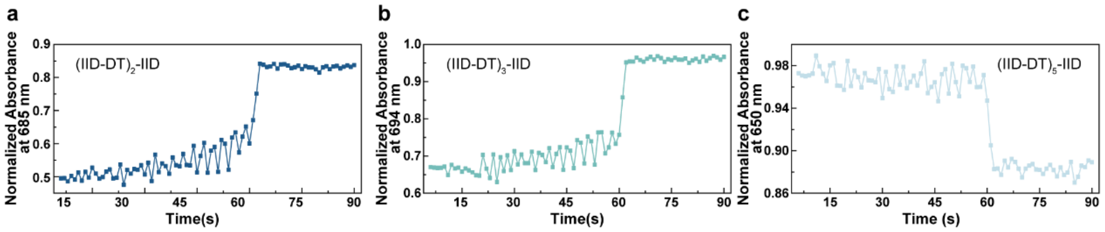

Supplementary Figure 23 | The absorbance variation of the oligomers during film formation process. (a) The normalized absorbance at 685 nm of $(IID\text{-}DT)_2$-IID during the *in situ* film formation kinetics. **(b)** The normalized absorbance at 694 nm of $(IID\text{-}DT)_3$-IID during the *in situ* film formation kinetics. **(c)** The normalized absorbance at 650 nm of $(IID\text{-}DT)_5$-IID during the *in situ* film formation kinetics.

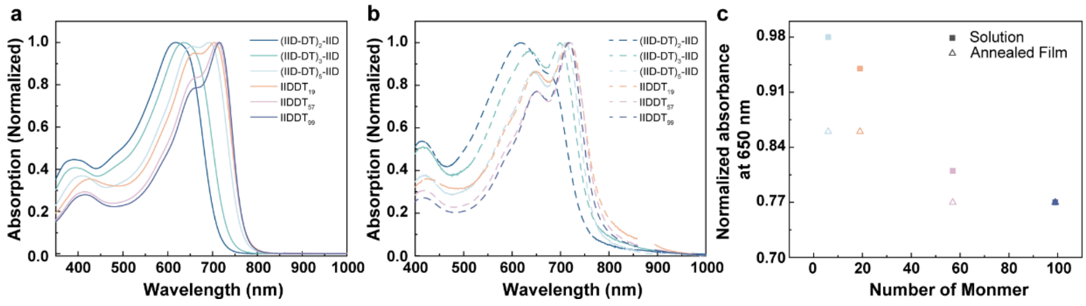

Supplementary Figure 24 | UV-*vis* absorption spectra of the samples. (a) UV-*vis* absorption spectra of $(IID\text{-}DT)_2$-IID, $(IID\text{-}DT)_3$-IID, $(IID\text{-}DT)_5$-IID, $IIDDT_{19}$, $IIDDT_{57}$, and $IIDDT_{99}$ solutions in *o*DCB. **(b)** UV-*vis* absorption spectra of annealed $(IID\text{-}DT)_2$-IID, $(IID\text{-}DT)_3$-IID, $(IID\text{-}DT)_5$-IID, $IIDDT_{19}$, $IIDDT_{57}$, and $IIDDT_{99}$ films. **(c)** The normalized absorbance at 650 nm of $(IID\text{-}DT)_5$-IID and these three polymer solutions and annealed films. Compared with solution spectra, there showed new characteristic absorptions at 680 nm and 698 nm of $(IID\text{-}DT)_2$-IID and $(IID\text{-}DT)_3$-IID film spectra, respectively, indicating the transform from isolation to aggregation of molecules.

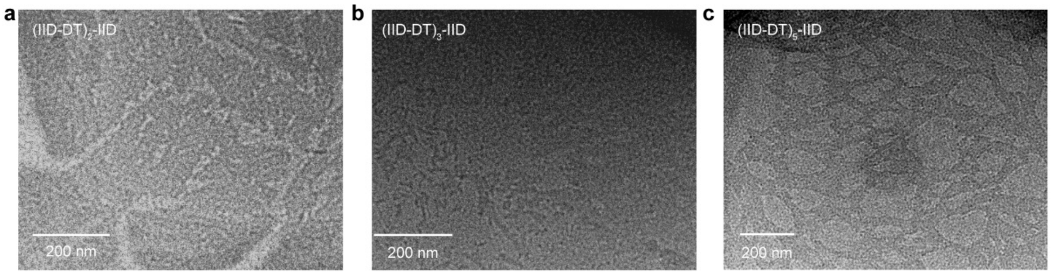

Supplementary Figure 25 | The EM images of sample films. (a) (IID-DT)$_2$-IID, **(b)** (IID-DT)$_3$-IID, and **(c)** (IID-DT)$_5$-IID films deposited from 1 g·L^{-1} *o*DCB solutions.

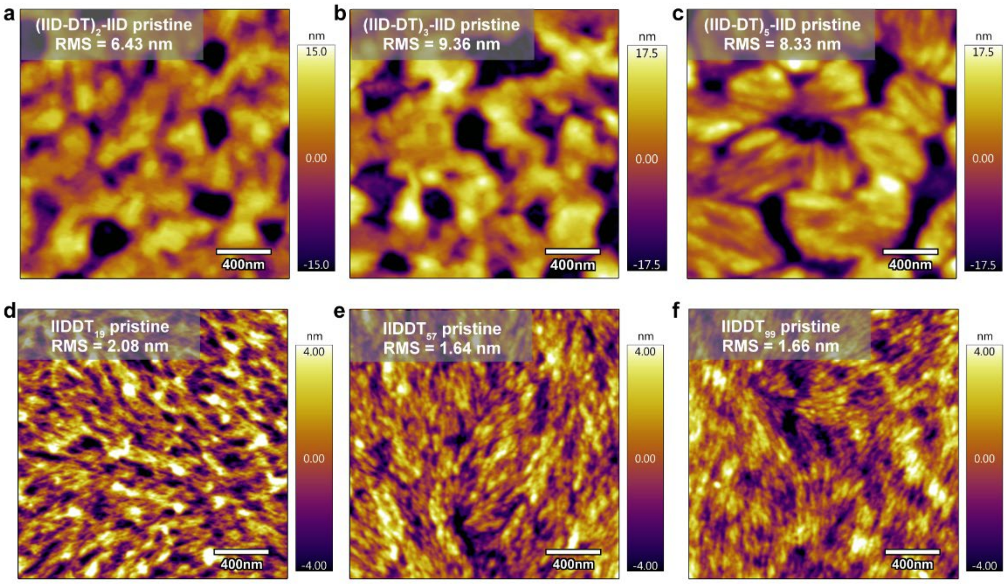

Supplementary Figure 26 | AFM height images of pristine films. (a) (IID-DT)$_2$-IID, **(b)** (IID-DT)$_3$-IID, **(c)** (IID-DT)$_5$-IID, **(d)** IIDDT$_{19}$, **(e)** IIDDT$_{57}$, and **(f)** IIDDT$_{99}$ films spin-coated by 5g·L^{-1} *o*DCB solutions.

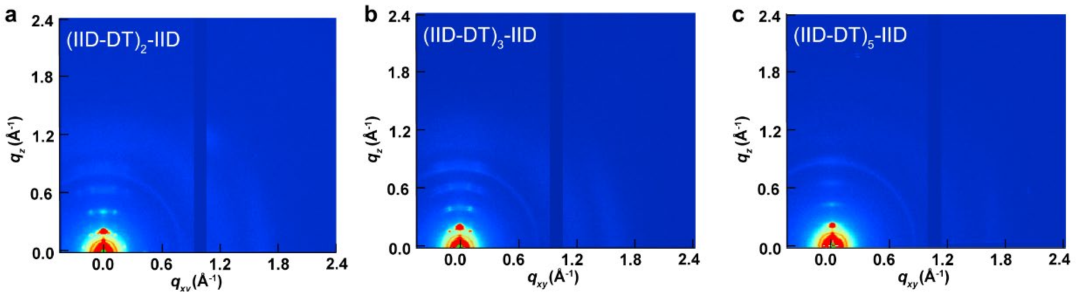

Supplementary Figure 27 | GIWAXS patterns of pristine films. (a) (IID-DT)$_2$-IID, **(b)** (IID-DT)$_3$-IID, and **(c)** (IID-DT)$_5$-IID.

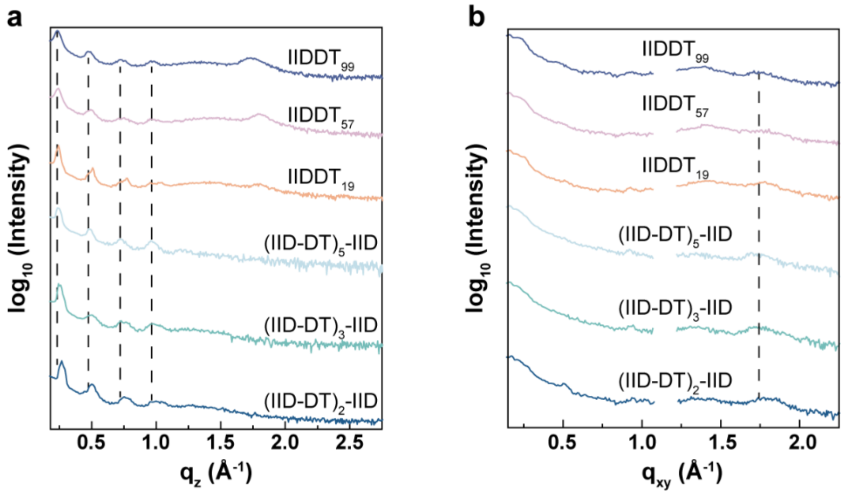

Supplementary Figure 28 | GIWAXS analysis. (a) Out-of-plane and **(b)** in-plane diffractions in GIWAXS analysis of pristine films.

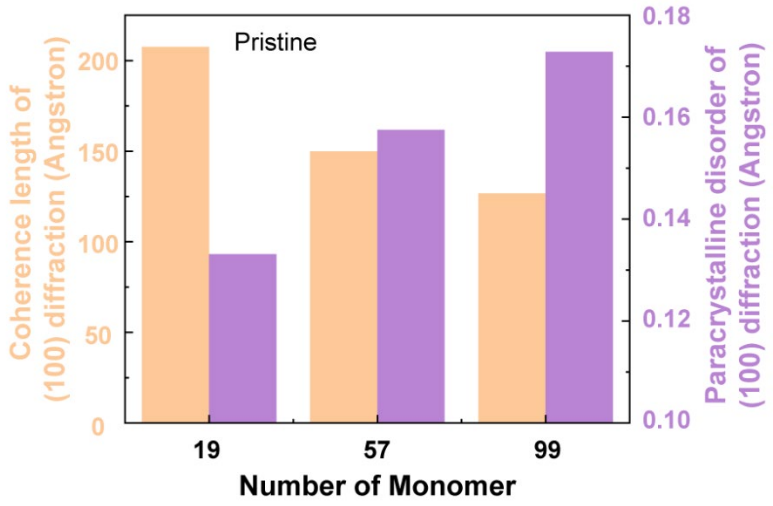

Supplementary Figure 29 | GIWAXS analysis. GIWAXS analysis on coherence length of (100) diffractions of pristine films. GIWAXS analysis on paracrystalline disorder of (100) diffractions of pristine films.

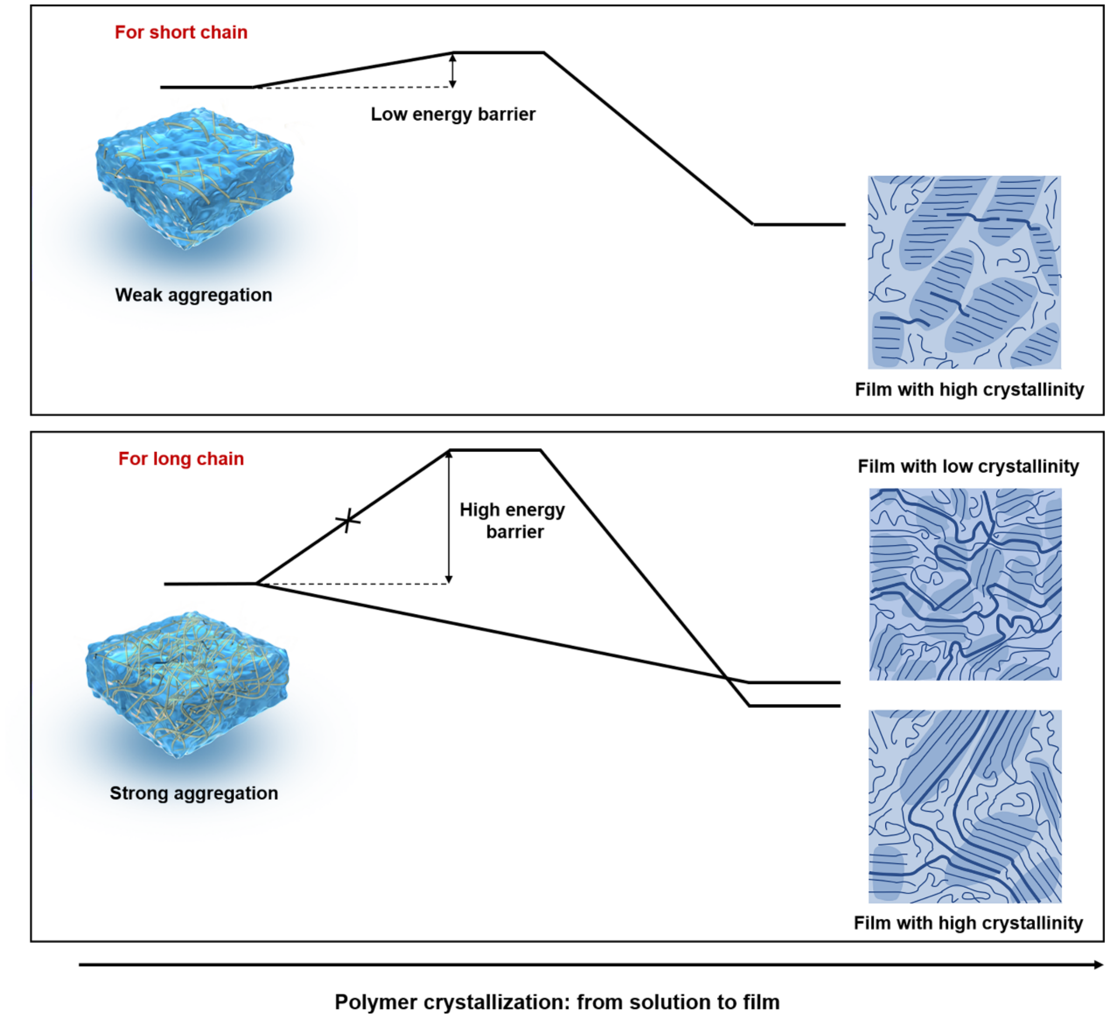

Supplementary Figure 30 | Schematic diagram of possible crystallization processes for polymers with different chain lengths. The schematics of film morphology (the right section) were adapted from ref.[3].

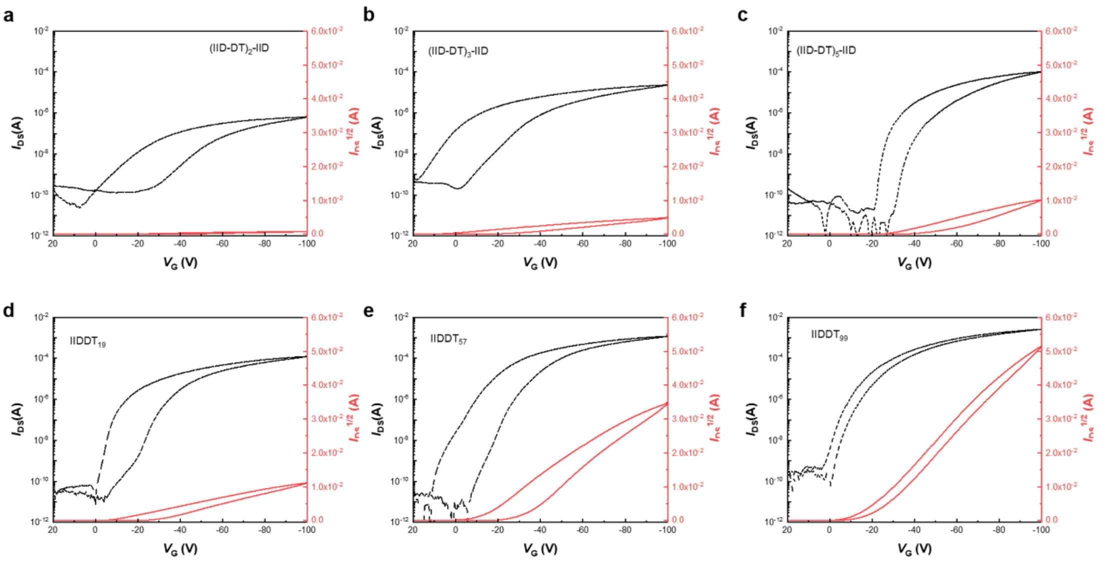

Supplementary Figure 31 | Charge transport performance. Representative transfer characteristics of **(a)** (IID-DT)$_2$-IID, **(b)** (IID-DT)$_3$-IID, **(c)** (IID-DT)$_5$-IID, **(d)** IIDDT$_{19}$, **(e)** IIDDT$_{57}$, and **(f)** IIDDT$_{99}$ transistors. The channel width and length are 1200 and 30 μm, respectively.

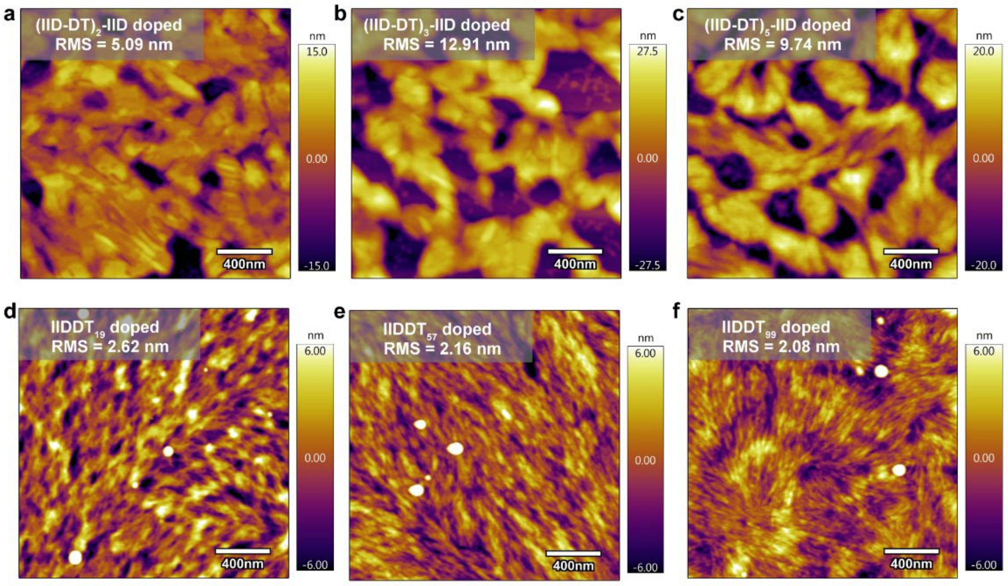

Supplementary Figure 32 | AFM height images of doped films. (a) (IID-DT)$_2$-IID, **(b)** (IID-DT)$_3$-IID, **(c)** (IID-DT)$_5$-IID, **(d)** IIDDT$_{19}$, **(e)** IIDDT$_{57}$, and **(f)** IIDDT$_{99}$ films doped by $FeCl_3$.

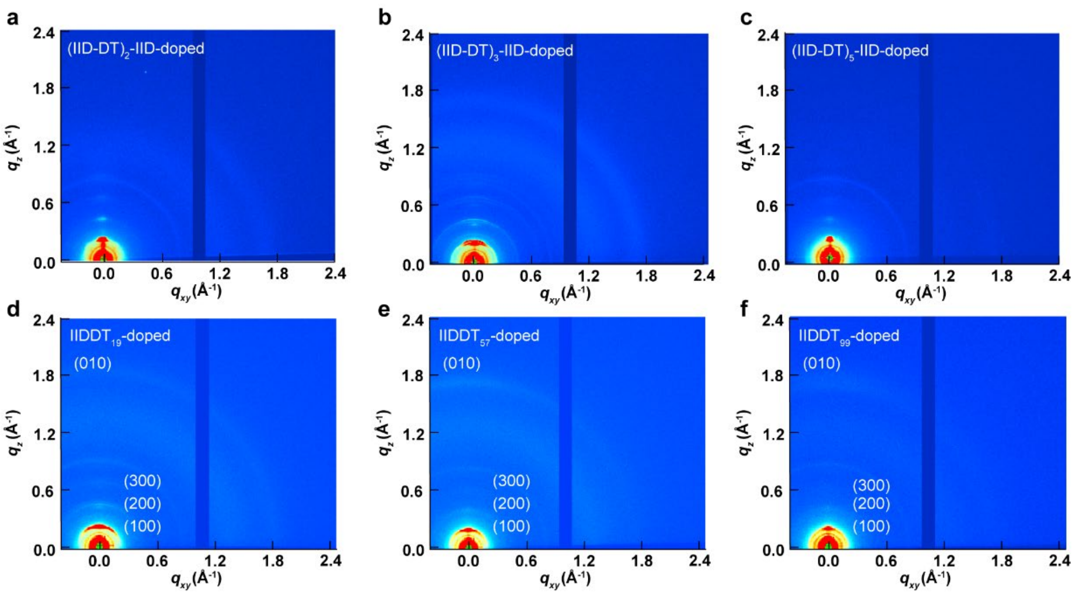

Supplementary Figure 33 | GIWAXS patterns of doped films. (a) (IID-DT)$_2$-IID, **(b)** (IID-DT)$_3$-IID, **(c)** (IID-DT)$_5$-IID, **(d)** IIDDT$_{19}$, **(e)** IIDDT$_{57}$, and **(f)** IIDDT$_{99}$ films doped by $FeCl_3$.

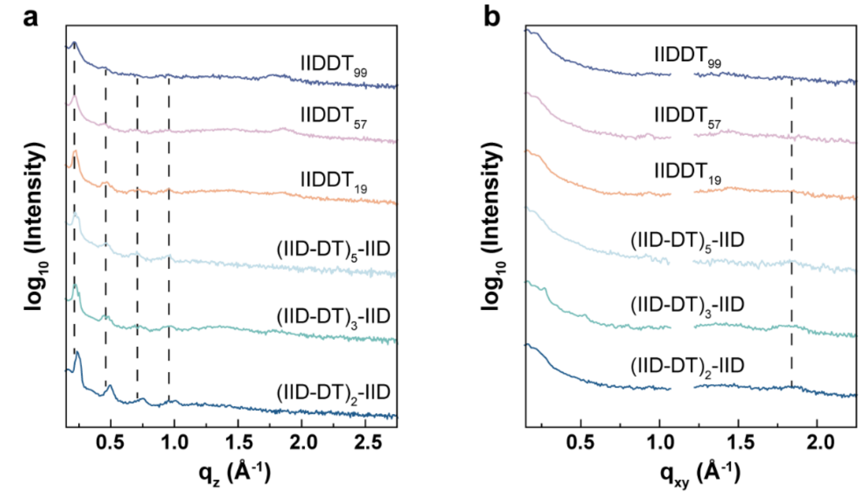

Supplementary Figure 34 | GIWAXS analysis. (a) Out-of-plane and **(b)** in-plane diffractions in GIWAXS analysis of doped films.

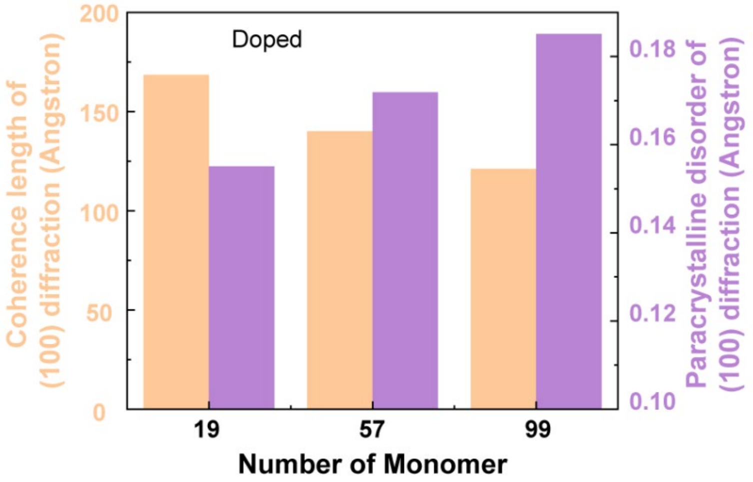

Supplementary Figure 35 | GIWAXS analysis. GIWAXS analysis on coherence length of (100) diffractions of doped films. GIWAXS analysis on paracrystalline disorder of (100) diffractions of doped films.

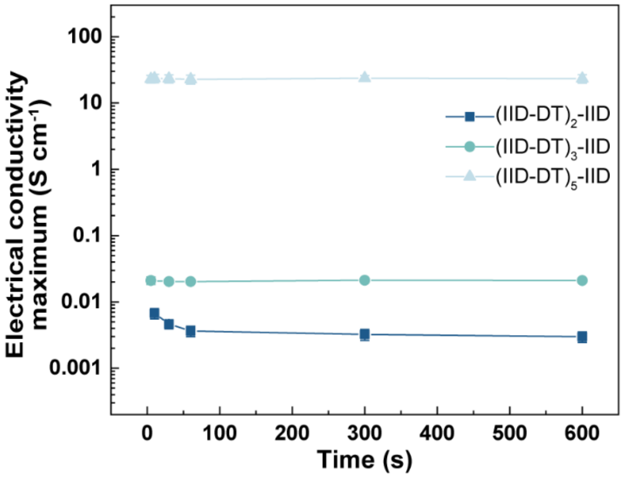

Supplementary Figure 36 | Electrical conductivities. Electrical conductivities of (IID-DT)$_2$-IID, (IID-DT)$_3$-IID and (IID-DT)$_5$-IID doped for 5, 10, 30, 60, 300, and 600 s, respectively. Each error bar was collected from more than 5 devices.

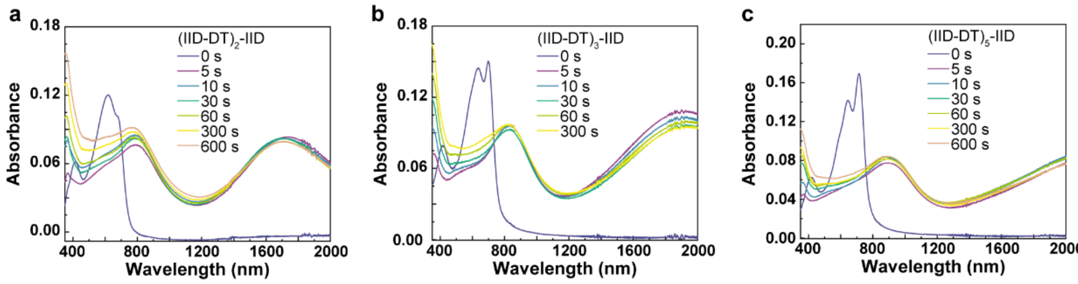

Supplementary Figure 37 | Absorption spectra of doped films. *Vis*-NIR absorption spectra of (**a**) (IID-DT)$_2$-IID, (**b**) (IID-DT)$_3$-IID, (**c**) (IID-DT)$_5$-IID films as a function of time (0, 5, 10, 30, 60, 300, and 600 s) under doping.

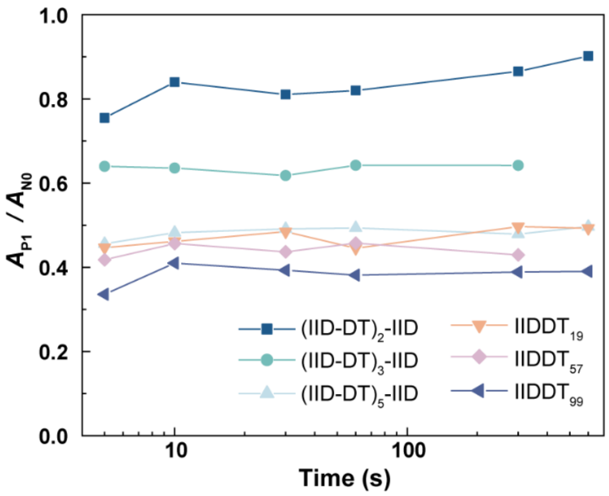

Supplementary Figure 38 | The analysis of absorption spectra. The ratios of A_{P1}/A_{N0} evaluated from the *Vis*-NIR absorption spectra of these doped oligomers and polymers. For (IID-DT)$_2$-IID, the N_0 peak was at 680 nm, the P_1 peak was at around 790 nm; For (IID-DT)$_3$-IID, the N_0 peak was at 699 nm, the P_1 peak was at around 833 nm; For (IID-DT)$_5$-IID and these three polymers, the N_0 peak was at 713 nm, the P_1 peak was at around 900 nm; A_{P1} was the absorbance of P_1 peak and A_{N0} was the intensity of neutral peak at 713 nm in pristine film.

Supplementary Table 1 | The lamellar distance and *π*-*π* stacking distance of pristine and doped films of these oligomers and polymers.

Pristine	Lamellar (Å)	*π*-*π* stacking (Å)	Doped	Lamellar (Å)	*π*-*π* stacking (Å)
$(IID\text{-}DT)_2$-IID	23.45	3.50	$(IID\text{-}DT)_2$-IID	26.23	3.42
$(IID\text{-}DT)_3$-IID	24.91	3.58	$(IID\text{-}DT)_3$-IID	28.39	3.45
$(IID\text{-}DT)_5$-IID	25.82	3.61	$(IID\text{-}DT)_5$-IID	27.95	3.44
$IIDDT_{19}$	25.97	3.54	$IIDDT_{19}$	28.62	/
$IIDDT_{57}$	26.25	3.52	$IIDDT_{57}$	29.23	/
$IIDDT_{99}$	26.71	3.58	$IIDDT_{99}$	29.31	/

Supplementary Table 2 | The purity of solvents and reagents commercially obtained in this work.

Reagent / Solvent	purity
6-bromoindoline-2,3-dione (compound **1**)	97%
2-([2,2'-bithiophen]-5-yl)-4,4,5,5-tetramethyl-1,3,2-dioxaborolane (compound **5**)	96%
chloroform	CCER
chlorobenzene	AR
o-dichlorobenzene	≥ 99%
1-chloronaphthalene	85%
nitromethane	CP

2. Supplementary NMR Spectra

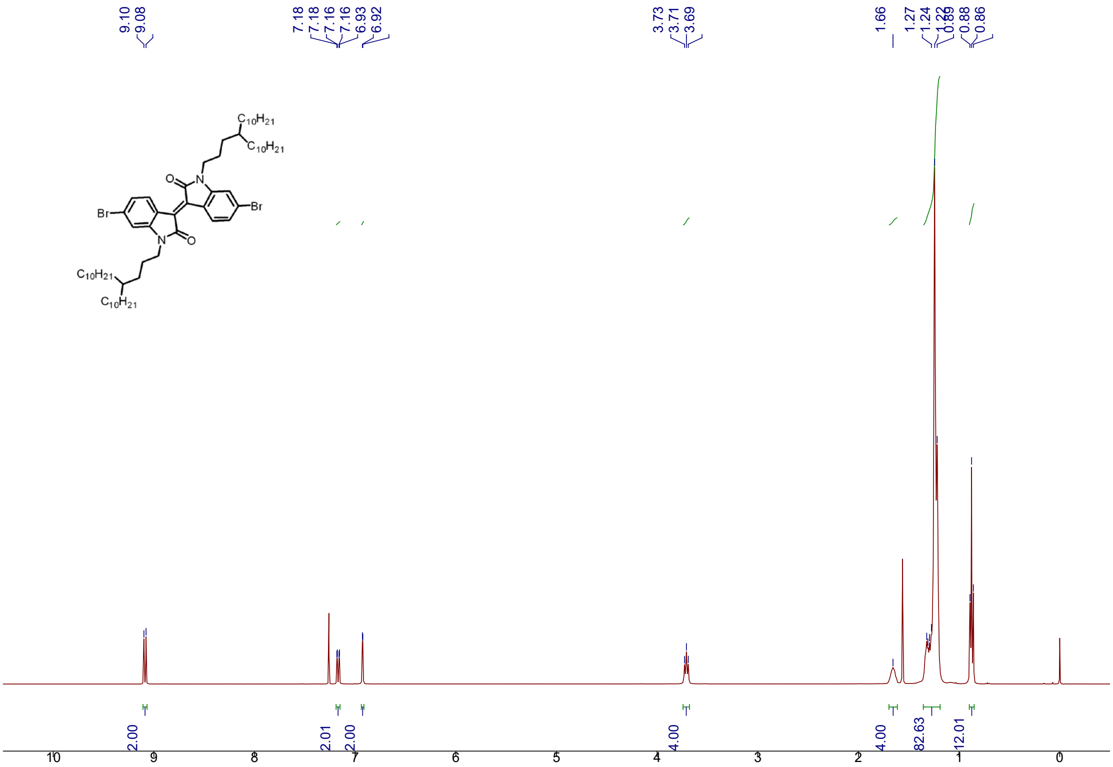

Supplementary Figure 39 | NMR spectrum. ^{1}H-NMR spectrum of **4** in $CDCl_3$ (298K, 400 MHz).

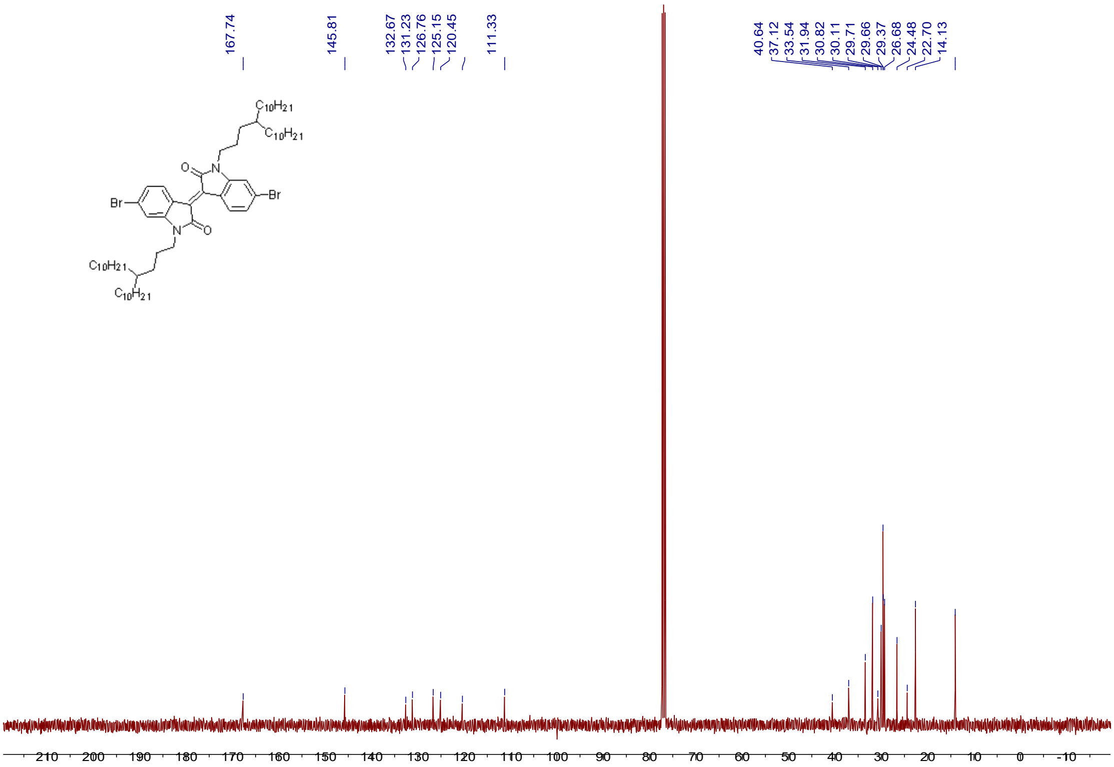

Supplementary Figure 40 | NMR spectrum. ^{13}C-NMR spectrum of **4** in $CDCl_3$ (298K, 101 MHz).

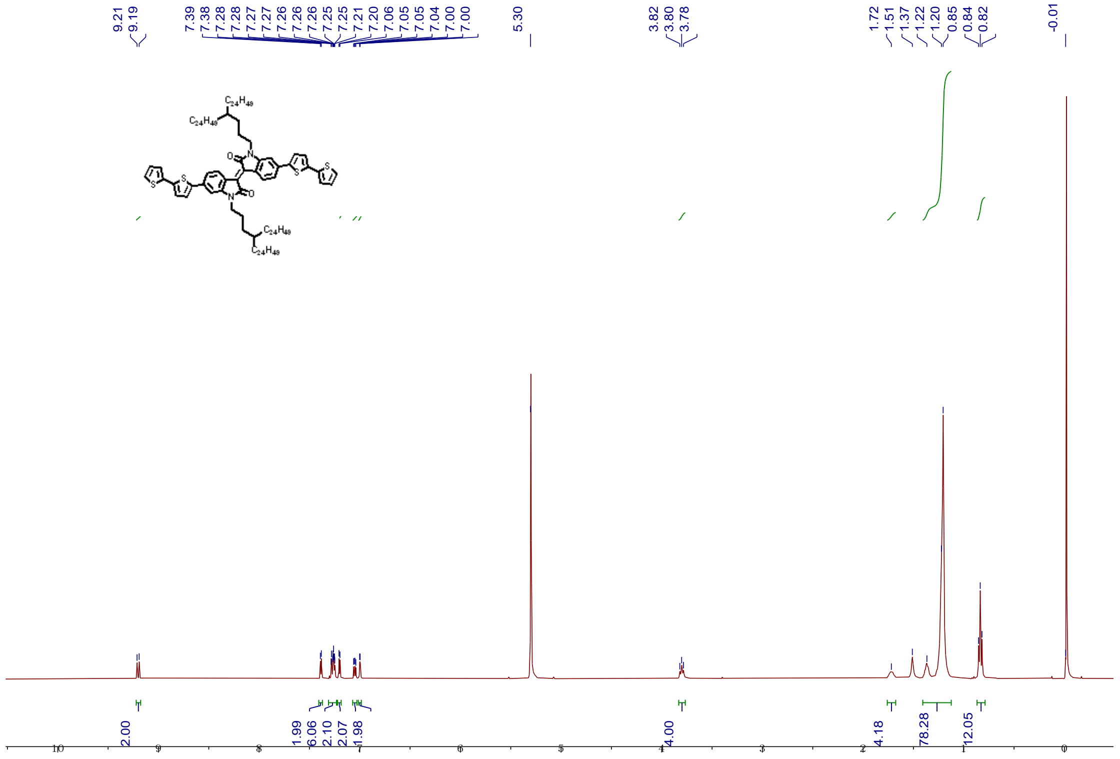

Supplementary Figure 41 | NMR spectrum. ^{1}H-NMR spectrum of **6** in CD_2Cl_2 (298K, 400 MHz).

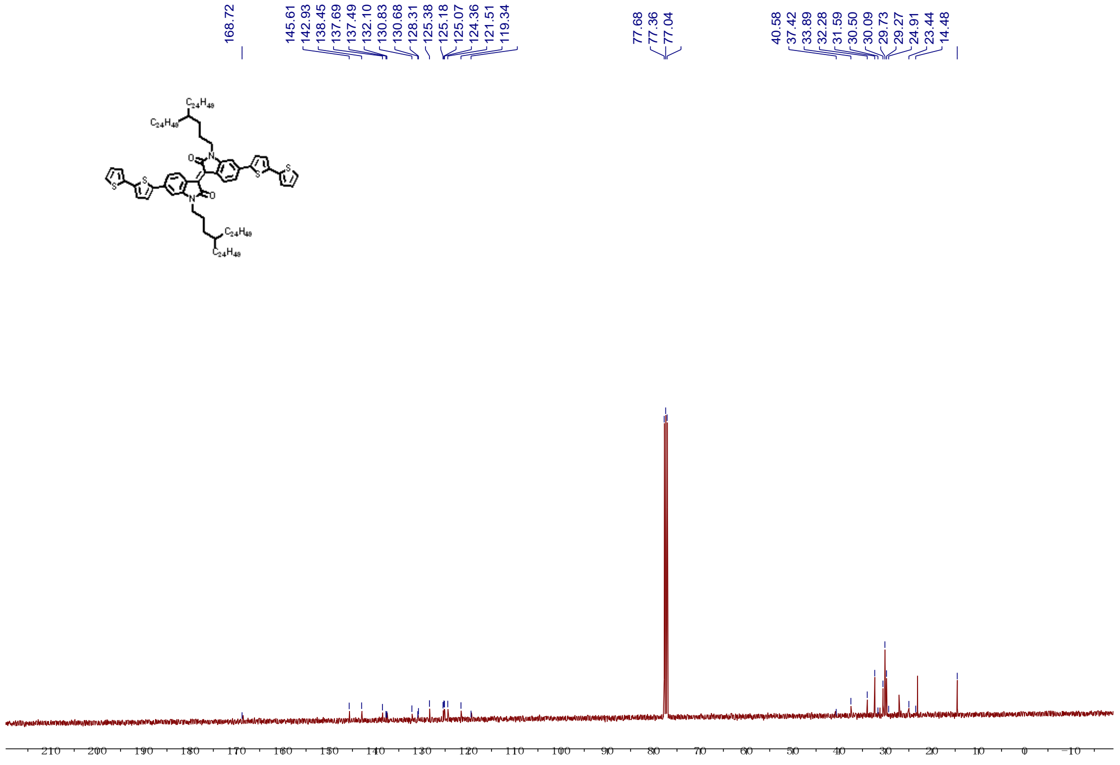

Supplementary Figure 42 | NMR spectrum. ^{13}C-NMR spectrum of **6** in $CDCl_3$ (298K, 101 MHz).

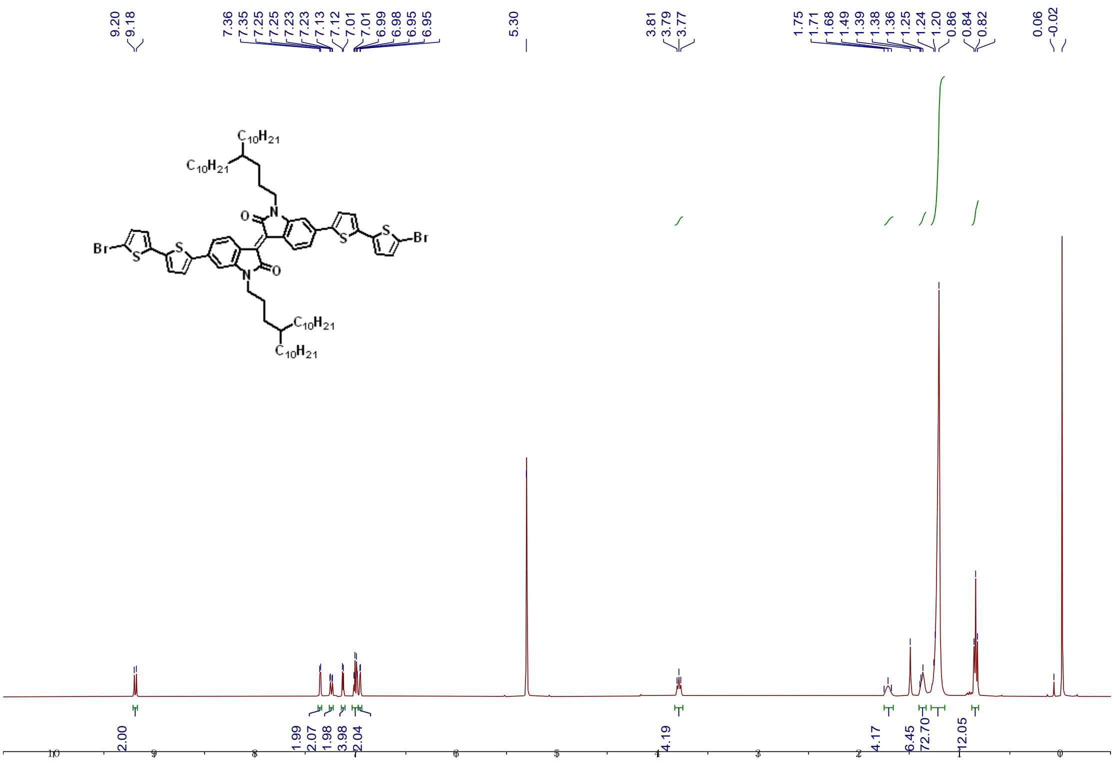

Supplementary Figure 43 | NMR spectrum. ^{1}H-NMR spectrum of **7** in CD_2Cl_2 (298K, 400 MHz).

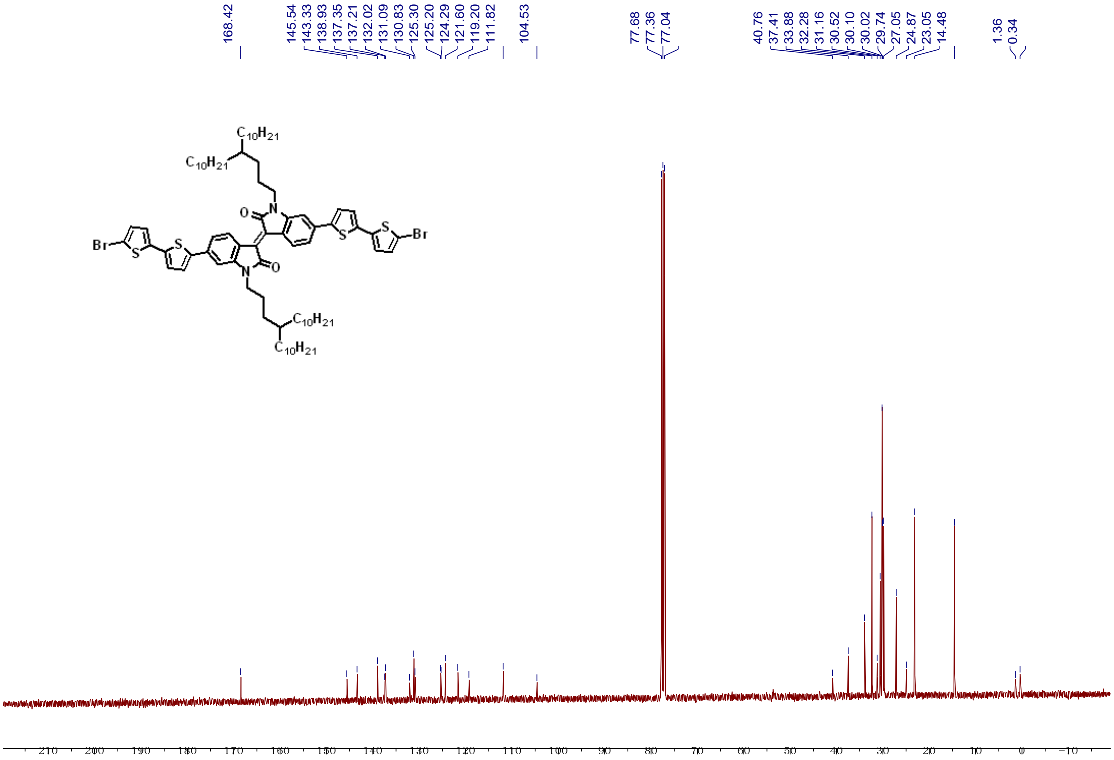

Supplementary Figure 44 | NMR spectrum. ^{13}C-NMR spectrum of **7** in $CDCl_3$ (298K, 101 MHz).

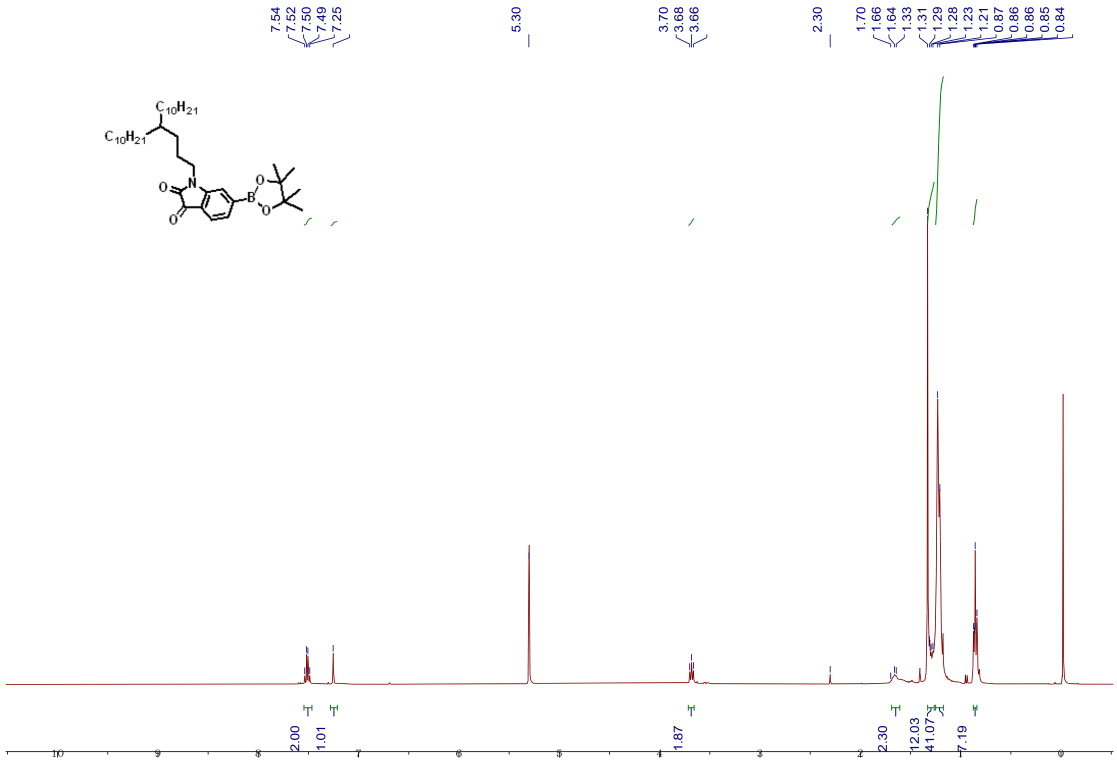

Supplementary Figure 45 | NMR spectrum. ^{1}H-NMR spectrum of **8** in CD_2Cl_2 (298K, 400 MHz).

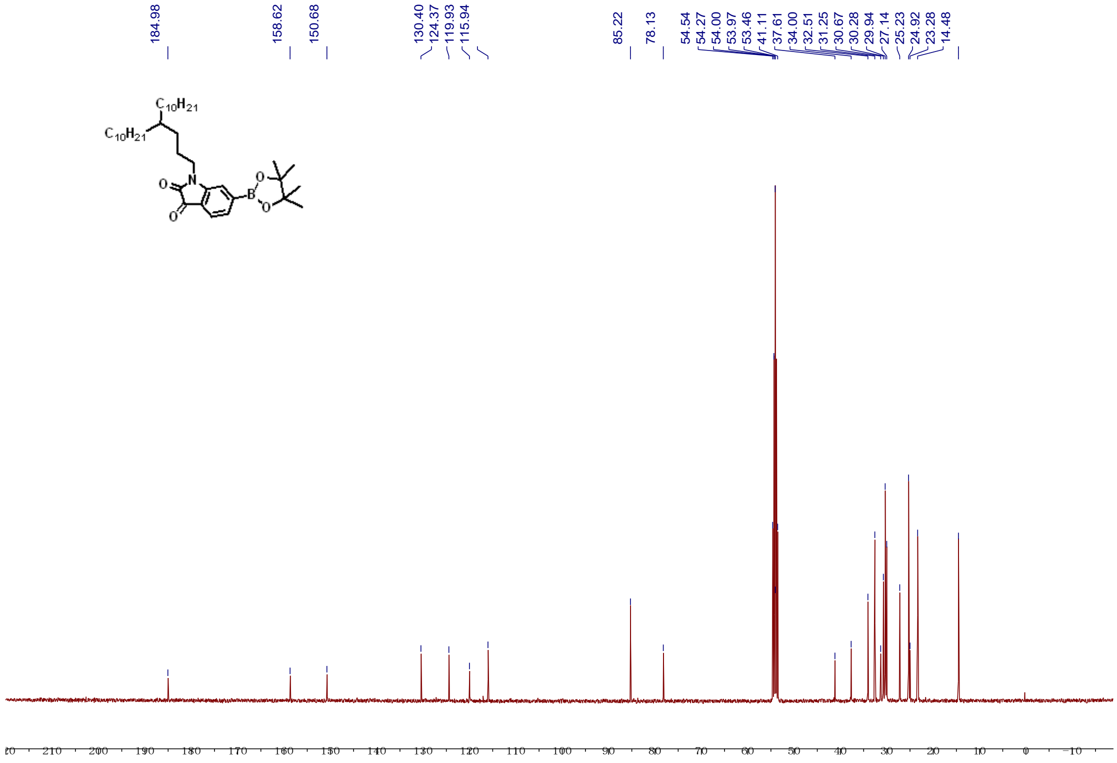

Supplementary Figure 46 | NMR spectrum. ^{13}C-NMR spectrum of **8** in $CDCl_3$ (298K, 101 MHz).

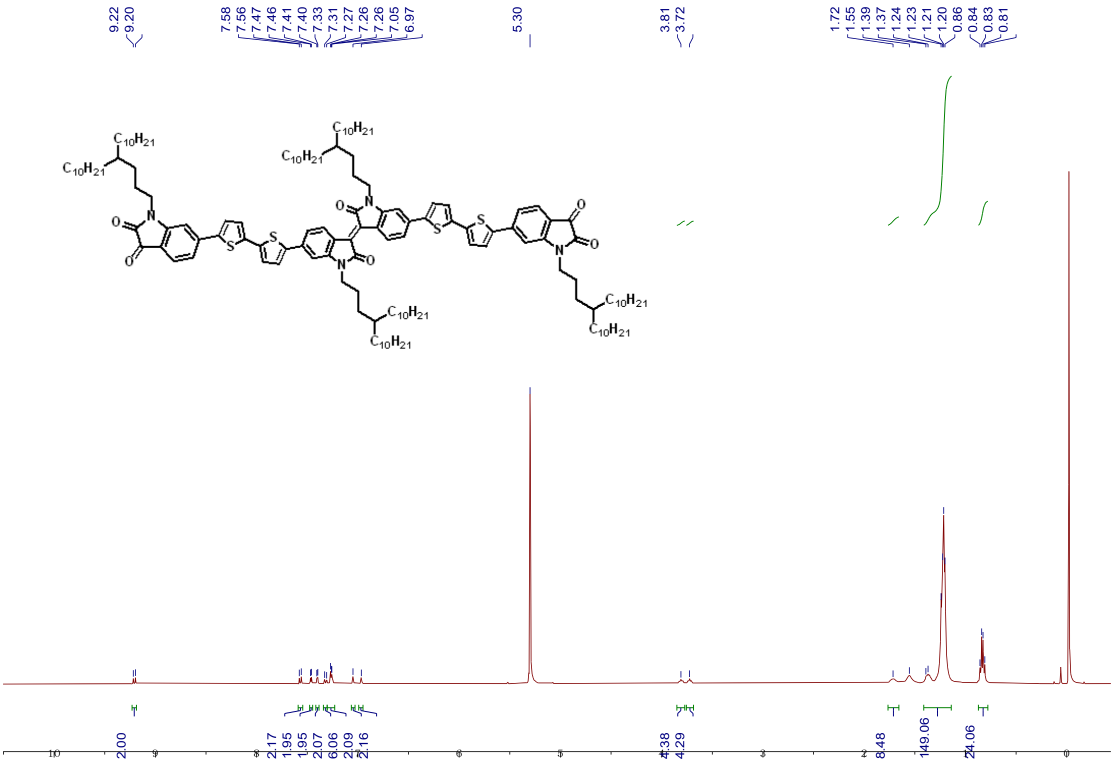

Supplementary Figure 47 | NMR spectrum. ^{1}H-NMR spectrum of **9** in CD_2Cl_2 (298K, 400 MHz).

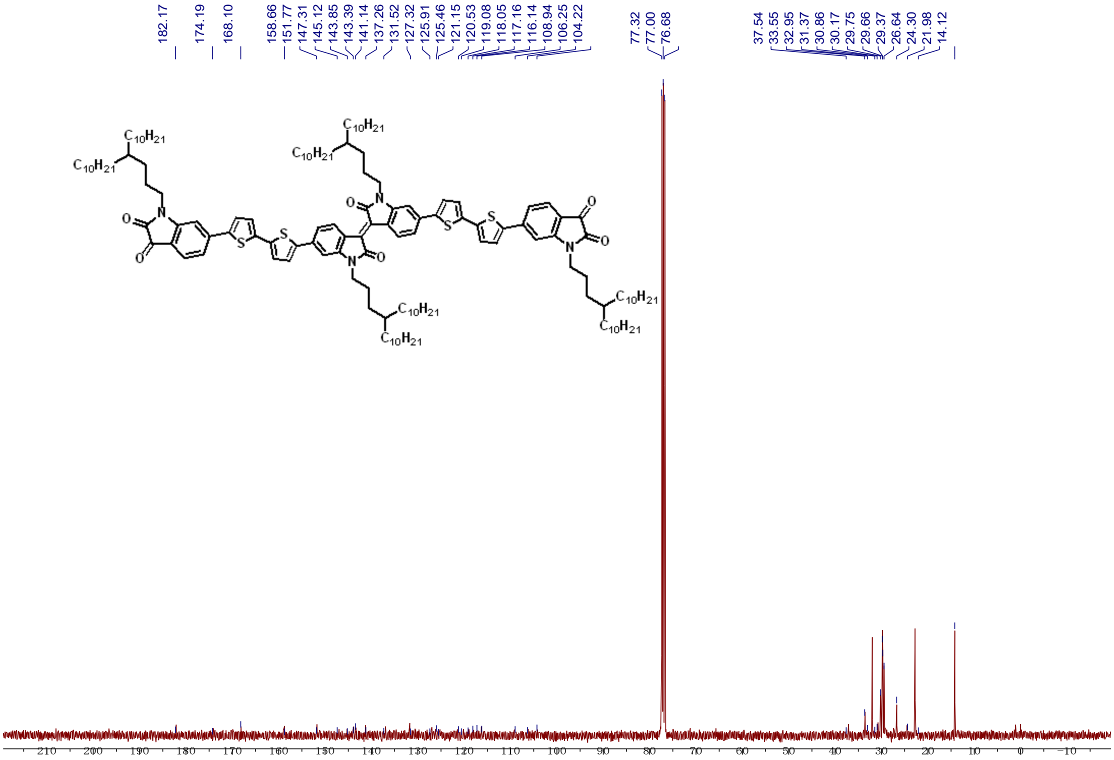

Supplementary Figure 48 | NMR spectrum. ^{13}C-NMR spectrum of **9** in $CDCl_3$ (298K, 101 MHz).

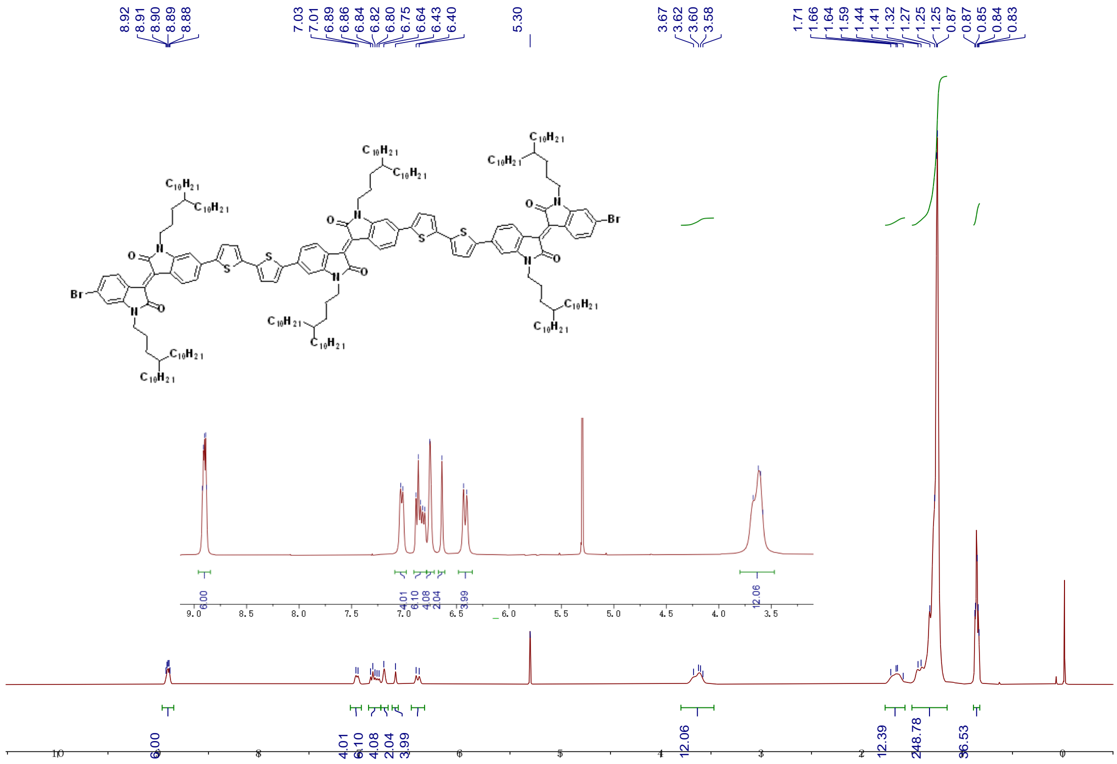

Supplementary Figure 49 | NMR spectrum. ^{1}H-NMR spectrum of **(IID-DT)$_2$-IID** in CD_2Cl_2 (298K, 400 MHz).

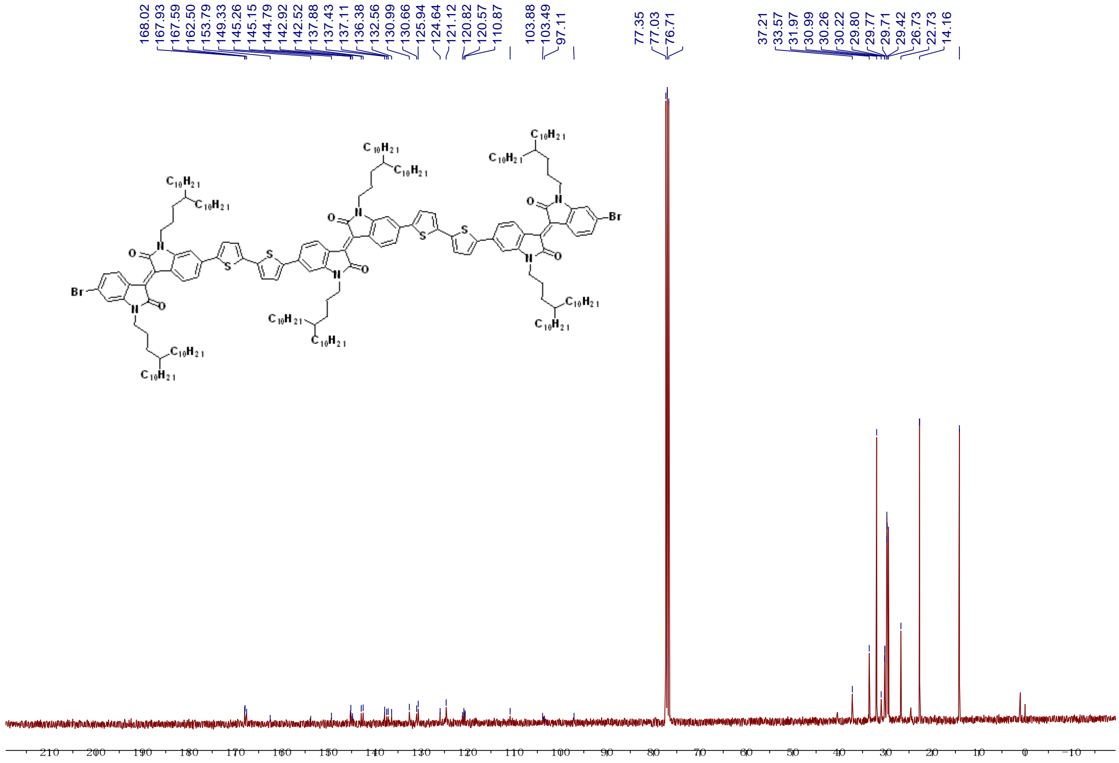

Supplementary Figure 50 | NMR spectrum. ^{13}C-NMR spectrum of **(IID-DT)$_2$-IID** in $CDCl_3$ (298K, 101 MHz).

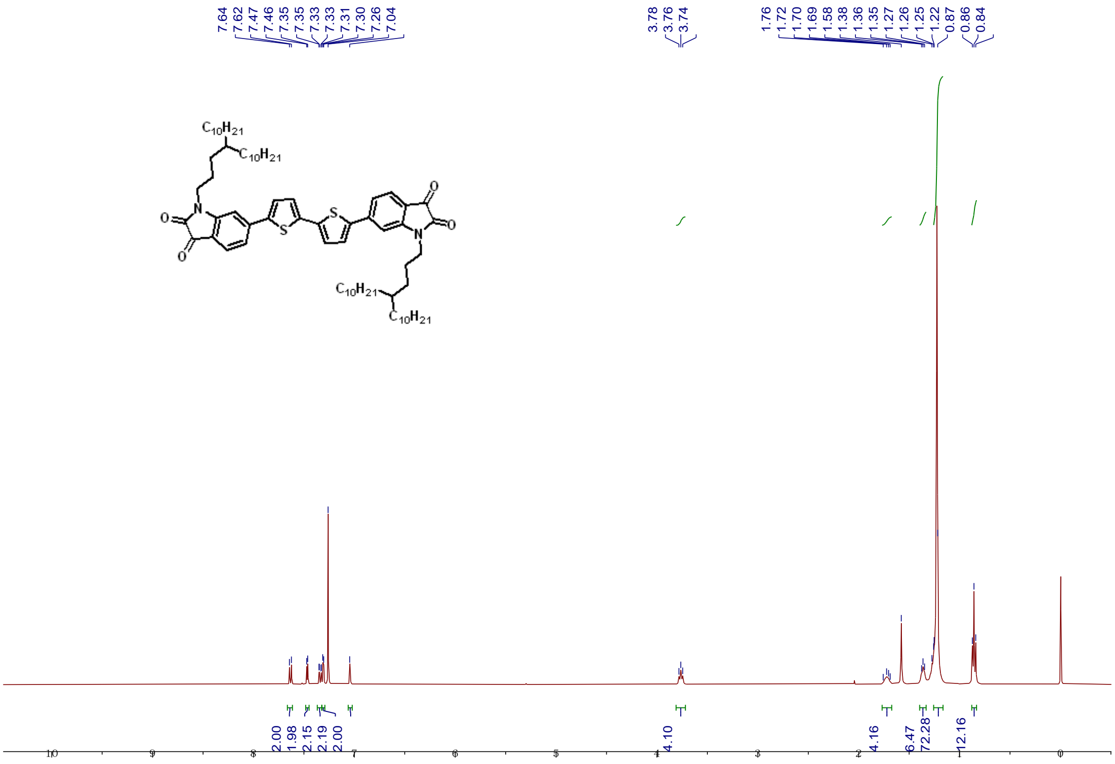

Supplementary Figure 51 | NMR spectrum. ^{1}H-NMR spectrum of **10** in $CDCl_3$ (298K, 400 MHz).

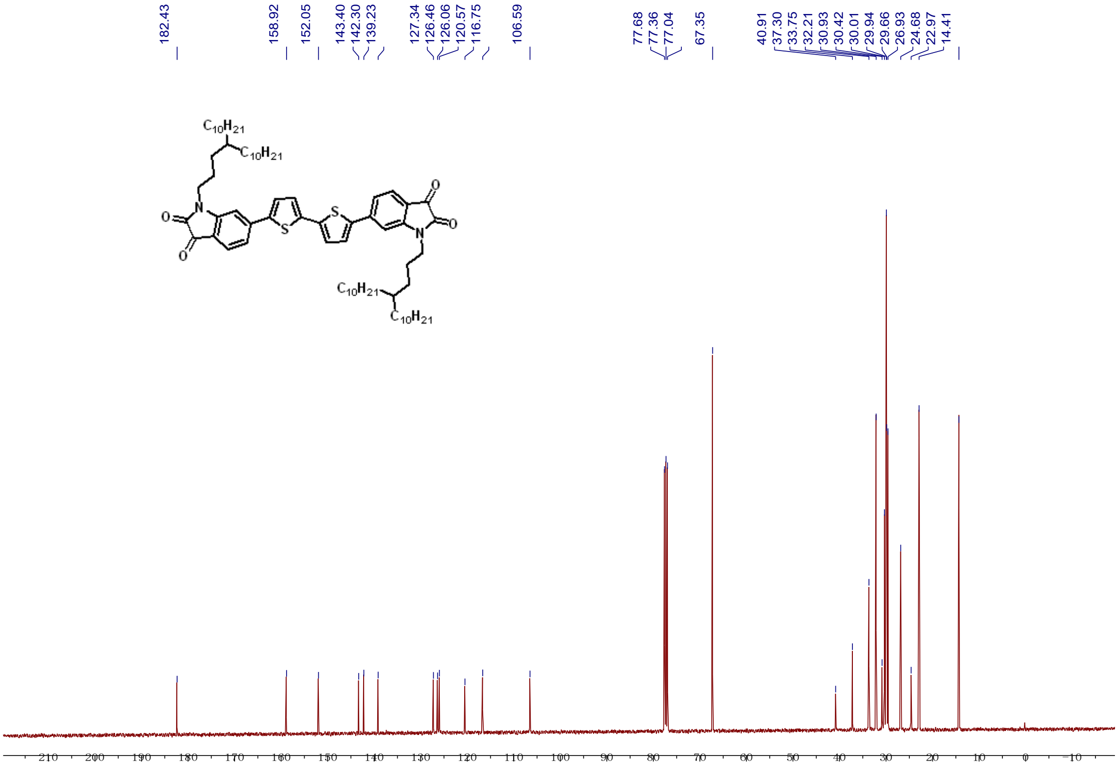

Supplementary Figure 52 | NMR spectrum. ^{13}C-NMR spectrum of **10** in $CDCl_3$ (298K, 101 MHz).

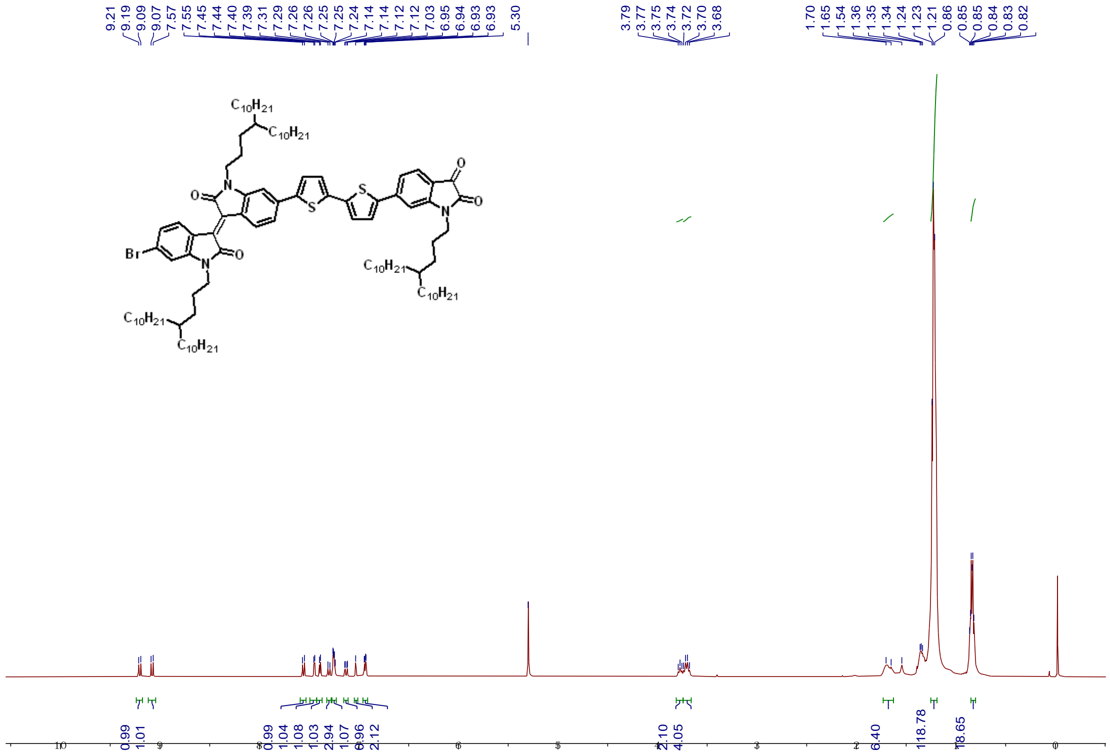

Supplementary Figure 53 | NMR spectrum. ^{1}H-NMR spectrum of **11** in $CDCl_3$ (298K, 400 MHz).

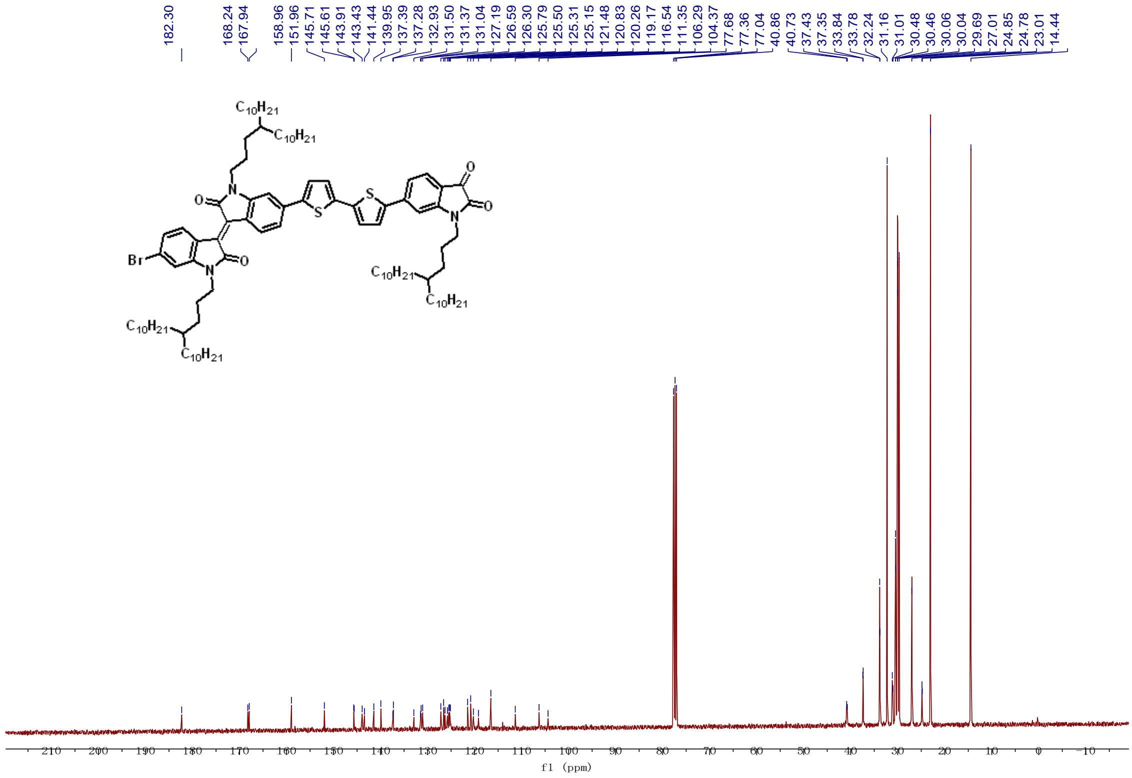

Supplementary Figure 54 | NMR spectrum. ^{13}C-NMR spectrum of **11** in $CDCl_3$ (298K, 101 MHz).

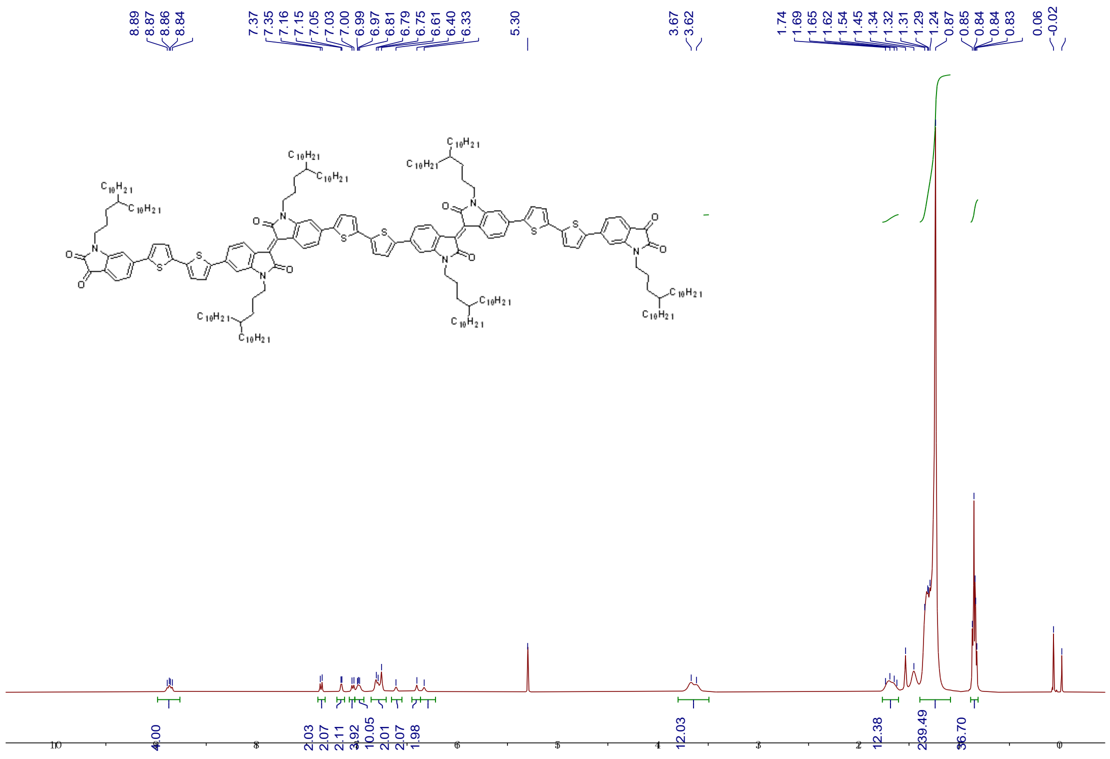

Supplementary Figure 55 | NMR spectrum. ^{1}H-NMR spectrum of **12** in $CDCl_3$ (298K, 400 MHz).

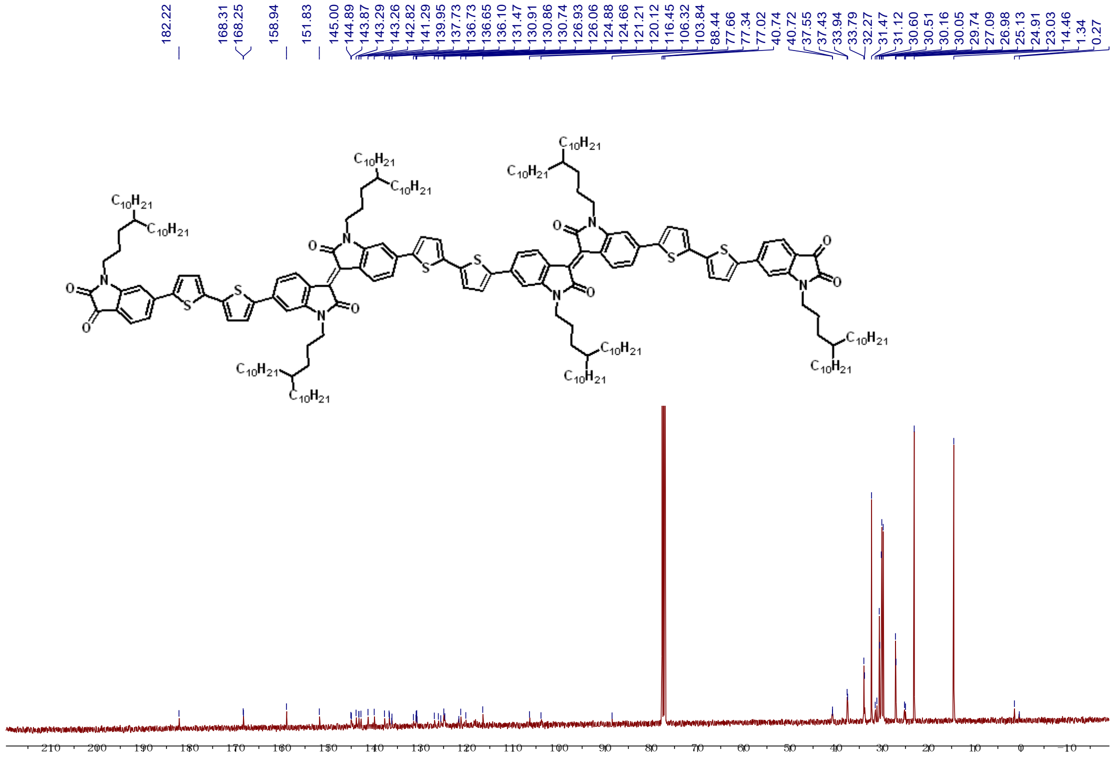

Supplementary Figure 56 | NMR spectrum. ^{13}C-NMR spectrum of **12** in $CDCl_3$ (298K, 101 MHz).

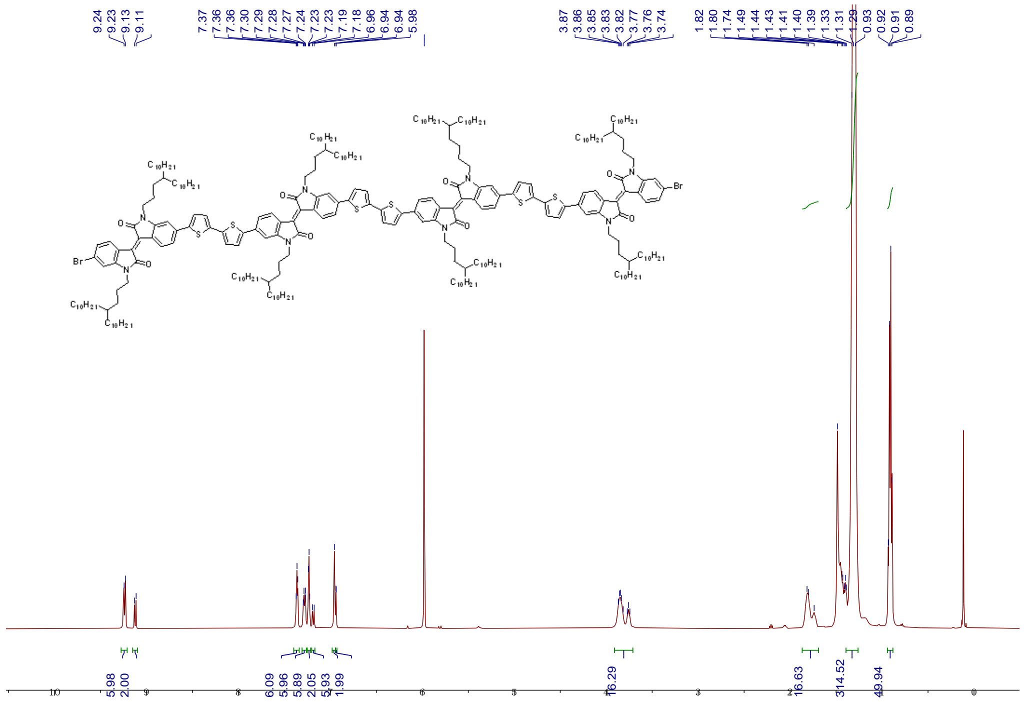

Supplementary Figure 57 | NMR spectrum. ^{1}H-NMR spectrum of **(IID-DT)$_3$-IID** in $C_2Cl_4D_2$ (363K, 500 MHz).

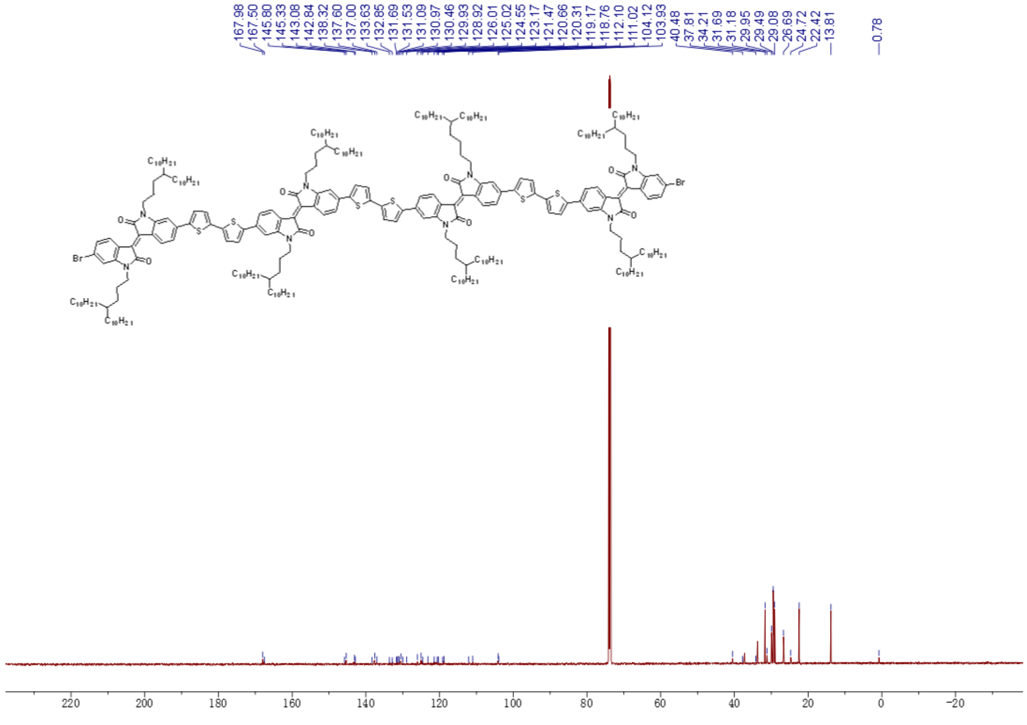

Supplementary Figure 58 | NMR spectrum. ^{13}C-NMR spectrum of **(IID-DT)$_3$-IID** in $C_2Cl_4D_2$ (363K, 126 MHz).

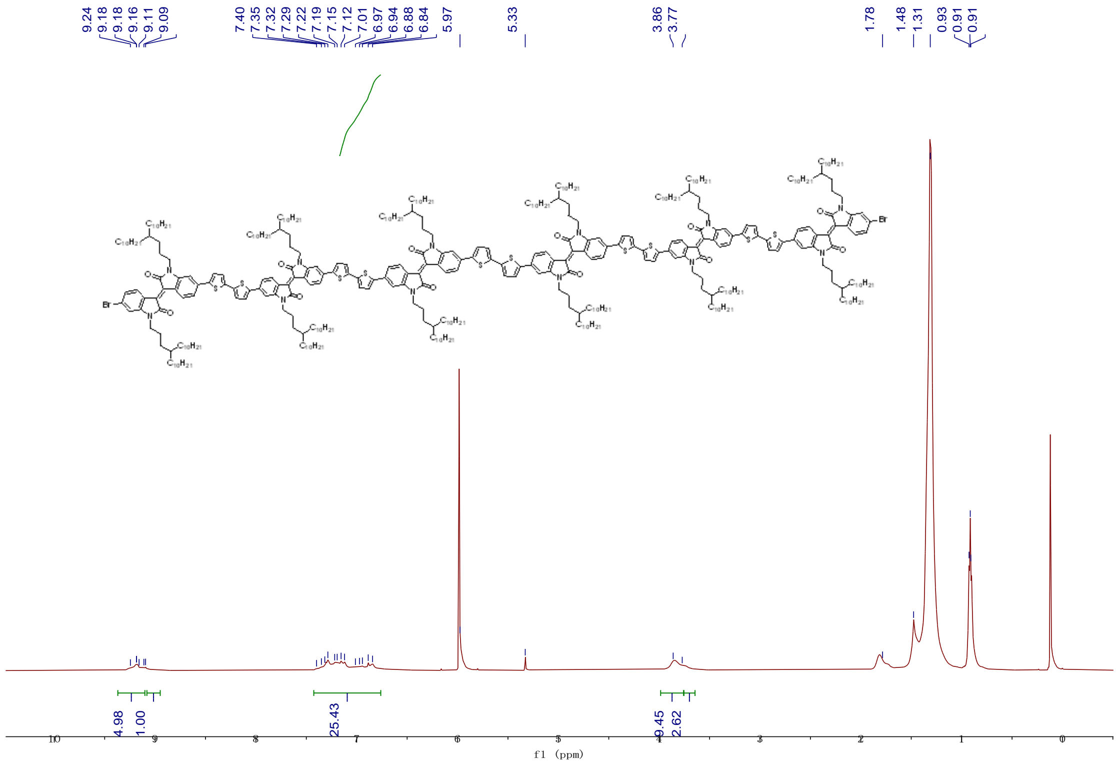

Supplementary Figure 59 | NMR spectrum. ^{1}H-NMR spectrum of **(IID-DT)$_5$-IID** in $C_2Cl_4D_2$ (363K, 500 MHz).

3. Supplementary Notes 1-5

Supplementary Note 1. The comparison of confined LP-EM environment with the actual situation of batch solution.

Indeed, the effect of surface interaction, confinement, and radiolysis in the liquid cell should be considered when interpret for the observed dynamics. The sluggish motion of nanoparticles and molecules in LP-EM compared to their behavior in bulk solution can be advantageous in capturing and analyzing intermediate states during a dynamic process. The presence of the confinement and surface interactions in the liquid cell effectively retard the kinetics of the system such that it acts as a “slow motion” camera, allowing to capture the intermediate states that are too transient to capture in bulk solution. However, it is important to carefully consider the potential artifacts of confinement and surface interactions on the interpretation of the observed behavior. The conclusions drawn from LP-EM experiments were carefully correlated with those from other experiments conducted in bulk solution. The gas-liquid interface plays a critical role in the film formation using solution processing method, which has a profound impact on the inheritance of solution-state aggregation into solid-state microstructures. This has been discussed in the main test and demonstrated by *in situ* UV-*vis* absorption spectra, AFM, and EM. Besides, the results analyzed from the temperature-dependent absorption spectra about the disaggregation process were also self-consistent with the results of LP-EM. With the conclusion from other analytical tools, we believe that the data generated by LP-EM experiments under current experimental conditions can reflect the disassembly process that occurs in batch solution as realistically as possible. Furthermore, it is noteworthy that the disassembly process induced by the liquid-gas interface, despite potential kinetic disparities in bulk solution, still serves as a representative model for comprehending the mechanisms of film formation. In this context, film formation involves interfacial phenomena, including surface tension and shear forces during the process of evaporation. Hence, we propose that the findings ascertained via LP-EM experiments conducted under the prevailing experimental conditions can reflect the disassembly process occurring in a batch solution.

Supplementary Note 2. The reversibility of aggregates’ disaggregation in graphene liquid cell (GLC)

According to literature, we believe that the onset of the disassembly process can be attributed to the surface tension and/or shear force at the gas-liquid interface, which overcomes the van der Waals forces arising from stacking in our system. However, if the gas-liquid interface gradually shrinks and the local concentration increases, we think that the chains have a possibility to reaggregate due to the disappearance of driven force, as shown in Supplementary Fig. 12.

Supplementary Note 3. The driven force of the disaggregation of conjugated polymers in LP-EM.

The onset of the disassembly process can be attributed to the surface tension and/or shear force at the gas-liquid interface[4], which overcomes the van der Waals forces arising from stacking in our system.

Supplementary Note 4. The relationship between the location of aggregates and the disassemble process.

Since there is only one bubble in the graphene liquid cell and the disassemble process occurs at the gas-liquid interface, we further analyzed the differences between polymer strands located at the concave interface and in the middle of the bubble (as shown in Supplementary Fig. 15a). We observed that there were insignificant differences in the changes of projected area between the

polymer strands located at the concave interface, leading us to believe that the disassemble process is not related to the location of the polymer strands at a single bubble in the liquid cell (Supplementary Fig. 15b). Therefore, we conclude that the dynamics we observed are independent of the location of the polymer strands and are instead governed by the local bubble size and curvature, which are directly related to the local stress applied to the polymer strands.

Supplementary Note 5. Electron dose of LP-EM.

The expected critical dose in liquids is theoretically presumed to be approximately 1000 $e^{-}\cdot Å^{-2}$, about two orders of magnitude higher than that in Cryo-EM (approximately 100 $e^{-}\cdot Å^{-2}$ for protein structure and approximately 10 $e^{-}\cdot Å^{-2}$ for active enzyme structure[5]. This can be attributed to the more diffusive and less reactive radicals present, as well as the radical scavenging ability of graphene[6]. This estimation is reasonable and supported by previous works concerning more fragile bio-macromolecule, which imaged DNA motion in GLC[7,8] and observed intactness of whole mammalian cells in a liquid[5].

In our system, the beam dose rates of Supplementary Movies 1, 2, and 3 were 14.5 $e^{-1}\cdot Å^{-2}\cdot s^{-1}$, 9.6 $e^{-1}\cdot Å^{-2}\cdot s^{-1}$, and 3.9 $e^{-1}\cdot Å^{-2}\cdot s^{-1}$. Experimentally, we have observed that the damage to polymer strands manifests as severe scission and a sharp decrease in projection area within a span of approximately two seconds (blue area in Supplementary Fig. 16), which is consistent with common degradation patterns in GLCs[9]. At 157.6 s, we noted the onset of severe scission when the electron dose reached approximately 2283 $e^{-}\cdot Å^{-2}$, followed by a marked reduction in projected area at 173.8 s. Based on the apparent disruption of assemble structure, we determined the critical dose to be around 2000 $e^{-}\cdot Å^{-2}$. We also assessed the time and electron dose before the appearance of bubbles in five additional liquid cells that contained identical solvents. The total electron dose before bubbles appeared ranged between 966 and 4793 $e^{-}\cdot Å^{-2}$, leading us to conclude that our polymers can withstand at least 1000 $e^{-}\cdot Å^{-2}$. Furthermore, we assessed the total electron dose of Supplementary Movies 1, 2, and 3, which were 116 $e^{-}\cdot Å^{-2}$, 557 $e^{-}\cdot Å^{-2}$, and 59 $e^{-}\cdot Å^{-2}$, respectively, and concluded that all analyzed polymer strands remained intact.

4. Supplementary References

1. Cao, Y. *et al.* N-Fused BDOPV: A tetralactam derivative as a building block for polymer field-effect transistors. *Chem. Commun.* **51**, 10514–10516 (2015).
2. Lei, T. *et al.* Systematic Investigation of Isoindigo-Based Polymeric Field-Effect Transistors: Design Strategy and Impact of Polymer Symmetry and Backbone Curvature. *Chem. Mater.* **24**, 1762–1770 (2012).
3. Noriega, R. *et al.* A general relationship between disorder, aggregation and charge transport in conjugated polymers. *Nat. Mater.* **12**, 1038–1044 (2013).
4. Bae, Y. *et al.* Conformation Dynamics of Single Polymer Strands in Solution. *Adv. Mater.* **2202353**, 2202353 (2022).
5. de Jonge, N., Houben, L., Dunin-Borkowski, R. E. & Ross, F. M. Resolution and aberration correction in liquid cell transmission electron microscopy. *Nat. Rev. Mater.* **4**, 61–78 (2019).
6. Cho, H. *et al.* The use of graphene and its derivatives for liquid-phase transmission electron microscopy of radiation-sensitive specimens. *Nano Lett.* **17**, 414–420 (2017).
7. Chen, Q. *et al.* 3D motion of DNA-Au nanoconjugates in graphene liquid cell electron microscopy. *Nano Lett.* **13**, 4556–4561 (2013).
8. Wang, H., Li, B., Kim, Y.-J., Kwon, O.-H. & Granick, S. Intermediate states of molecular self-assembly from liquid-cell electron microscopy. *Proc. Natl. Acad. Sci. U. S. A.* **117**, 1283–1292 (2020).
9. Wang, H., Xu, Z., Mao, S. & Granick, S. Experimental Guidelines to Image Transient Single-Molecule Events Using Graphene Liquid Cell Electron Microscopy. *ACS Nano* **16**, 18526–18537 (2022).
