# Supplementary material for: Visualizing the multi-level assembly structures of conjugated molecular systems with chain-length dependent behavior
Source: Nat Commun. 2023 Jun 7;14:3340. doi: 10.1038/s41467-023-39133-w (PMC10247739; doi:10.1038/s41467-023-39133-w)
Supplement: Supplementary file 3 — Description of Additional Supplementary Files [file 41467_2023_39133_MOESM3_ESM.pdf]

Description of Additional Supplementary Files

File Name: Supplementary Movie 1
Description: The movie showed the dynamic disaggregation process of IIDDT19 in a liquid pocket. The images are captured with an exposure time of 0.1592 s per frame with 0.1592 s time difference between successive frames. Rolling average of consecutive five frames was applied to the image series to average out noises in the background.

File Name: Supplementary Movie 2
Description: In order to prove that the process of Movie 1 was not an incidental phenomenon, more samples were experiment and Movie 2 was gained. This movie also showed the captured dynamic disaggregation process of $IIDDT_{19}$ in a liquid pocket. As shown in the movie, large aggregates also rapidly disaggregated into dispersed small aggregates. Several images were extracted as shown below. Images are captured with an exposure time of 0.5 s per frame with 1.5s time difference between successive frames.

File Name: Supplementary Movie 3
Description: This movie showed the captured dynamic disaggregation process of $(IID\text{-}DT)_{5\text{-}}IID$ in a liquid pocket. Similar with $IIDDT_{19}$, the disaggregation process of $(IID\text{-}DT)_{5\text{-}}IID$ also experience a transformation from a large aggregate to medium aggregates and finally to dispersed small aggregates, as marked by red circle. This also proved that the chains with length around 6 units have already formed aggregation structure in solution, which was consistent with the results of the temperature-dependent absorption spectra. Several images were extracted as shown below. These images are captured with an exposure time of 0.1592 s per frame with 0.1592 s time difference between successive frames.
